# BioCode: Two biologically compatible Algorithms for embedding data in non-coding and coding regions of DNA

**DOI:** 10.1186/1471-2105-14-121

**Published:** 2013-04-09

**Authors:** David Haughton, Félix Balado

**Affiliations:** 1 School of Computer Science and Informatics, University College Dublin, Belfield, Co. Dublin, Ireland

## Abstract

**Background:**

In recent times, the application of deoxyribonucleic acid (DNA) has diversified with the emergence of fields such as DNA computing and DNA data embedding. DNA data embedding, also known as DNA watermarking or DNA steganography, aims to develop robust algorithms for encoding non-genetic information in DNA. Inherently DNA is a digital medium whereby the nucleotide bases act as digital symbols, a fact which underpins all bioinformatics techniques, and which also makes trivial information encoding using DNA straightforward. However, the situation is more complex in methods which aim at embedding information in the genomes of living organisms. DNA is susceptible to mutations, which act as a noisy channel from the point of view of information encoded using DNA. This means that the DNA data embedding field is closely related to digital communications. Moreover it is a particularly unique digital communications area, because important biological constraints must be observed by all methods. Many DNA data embedding algorithms have been presented to date, all of which operate in one of two regions: non-coding DNA (ncDNA) or protein-coding DNA (pcDNA).

**Results:**

This paper proposes two novel DNA data embedding algorithms jointly called BioCode, which operate in ncDNA and pcDNA, respectively, and which comply fully with stricter biological restrictions. Existing methods comply with some elementary biological constraints, such as preserving protein translation in pcDNA. However there exist further biological restrictions which no DNA data embedding methods to date account for. Observing these constraints is key to increasing the biocompatibility and in turn, the robustness of information encoded in DNA.

**Conclusion:**

The algorithms encode information in near optimal ways from a coding point of view, as we demonstrate by means of theoretical and empirical (*in silico*) analyses. Also, they are shown to encode information in a robust way, such that mutations have isolated effects. Furthermore, the preservation of codon statistics, while achieving a near-optimum embedding rate, implies that BioCode pcDNA is also a near-optimum first-order steganographic method.

## Background

The potential of deoxyribonucleic acid (DNA) for use as a storage medium of digital data was realised just over a decade ago [1]. Many promising applications of this emerging field have been proposed, such as long term data storage [2] and genetic tagging [3]. It is likely that, with advancements in DNA sequencing and synthesising technologies, information embedding in the genome of living organisms will be routine in the near future. To date several data embedding algorithms have been proposed [1,2,4-8]. However, as we will see later, none of them fully comply with some recently highlighted biological restrictions. Not adhering to these restrictions can potentially be detrimental to the organism hosting the artificial information-carrying DNA. Here we propose two novel algorithms jointly called BioCode, which, unlike any previous ones, produce information-encoded DNA more biologically compatible for the host organism, thus improving the robustness of the encoded message. In addition to operating under strict constraints, never dealt with before, they encode information in near optimal ways. This is to the extent that for one such algorithm the embedding rate (in information bits embedded per DNA component) is indistinguishable from the optimal theoretical bound.

Interest in using DNA for information storage (genetic memories) is growing, not surprisingly, as it is a highly compact and potentially durable medium with the ability to make replicas of information costing little energy. Stored information is passed from generation to generation when placed anywhere in the genome of asexual organisms. Data encoded in DNA is subject to errors caused by random mutations in the organism’s DNA, but if encoded correctly it may still be retrievable after millions of generations or more [7]. Encoding information in sexually reproducing organisms is more complicated due to the effects of genetic crossover. However this issue has been tackled by Heider et. al [9], who proposed embedding information in mitochondrial DNA (mtDNA). In most sexually reproducing species mtDNA is inherited from the mother alone, making it an ideal location for data embedding.

Another application of robust DNA data embedding algorithms is the genetic tagging of organisms. This would be of interest to individuals researching and working with artificial or genetically modified organisms, allowing them to embed “ownership watermarks”. This was the case in one recent, high profile experiment performed by the J Craig Venter Institute (JCVI). A watermarked DNA sequence, representing the researchers’ initials, was embedded in a chemically synthesized bacterial genome [10]. A further proposal is the application of DNA data embedding for tagging potentially hazardous viruses [11]. Unique watermarks could identify different laboratories handling viruses, and thus it would be possible to refute claims that some particular institution is the source of a viral outbreak.

Despite the different potential applications of DNA data embedding, all embedding algorithms should be designed based on some common principles. Many of the prior algorithm proposals have been made by researchers concerned primarily with the biological aspects of embedding an artificial DNA sequence, but which paid relatively little attention to the coding aspects of the problem. Instead we have designed the BioCode algorithms keeping in mind not only more stringent biological constraints, but also principles from digital communications. Firstly, the information-carrying DNA sequence should not hinder the host organisms’ development (that is, it should be as biocompatible as possible). Secondly, the embedded data should be retrievable as close as possible to a theoretical threshold (Shannon’s capacity), determined by the number of generations a message has been transmitted along and the mutation rate between generations. Finally, the algorithms should make economical use of DNA in terms of data storage, that is, maximise the embeddable payload for a given sequence length. We will demonstrate these properties through an *in silico* empirical analysis, in conjunction with theoretical estimates of achievable embedding rate.

There exist two distinct regions within the genomes of living organisms: protein-coding (pcDNA) regions and non-protein coding (ncDNA) regions. In the past, ncDNA was thought to have no function, however recent research suggests that up to 80% of ncDNA may be responsible for regulatory functions [12]. In the remaining 20% of ncDNA it is safe to assume that DNA can be freely overwritten. Indeed several authors have performed successful data embedding experiments *in vivo* in these regions [5,6]. The ncDNA data embedding algorithm we propose here is also designed to operate in this non functional 20% of ncDNA.

On the other hand pcDNA regions are responsible for the encoding of proteins, which are the basic building blocks of life. It is possible to modify pcDNA regions to encode information; however the constraints which an algorithm must operate under are more restrictive. The goal of each of the two BioCode algorithms presented here is to optimally embed information within each of the two types of DNA regions that we have discussed.

### Prior art

The DNA data embedding field was born a little over a decade ago with the seminal paper by Clelland et al. [1], in which the authors proposed and implemented a data embedding scheme. Alphanumeric data was embedded using a trivial assignment of base groupings to characters. The synthesised DNA in this case was embedded *in vitro*, but not sub-cloned into an organism’s genome. The work of Clelland et al. was built upon by Wong et al. [2], in which they performed *in vivo* embedding of data in bacterial ncDNA regions. Similar to Clelland et al’s encoding scheme, a base to alphanumeric translation table was used. Two bacteria were selected for embedding, *E. coli* and *D. radiodurans*. The latter has the ability to survive in harsh environments such as those containing high levels of ionizing radiation, implying that the encoded message would also be resilient under such conditions.

The first paper to discuss error correction for information encoded in DNA was by Smith *et al*[13]. Since any information embedded in DNA is replicated from generation to generation, any difference between encoded information may be resolved by examining copies obtained from different organisms. Also, there exists genetic machinery in the cell which maintains DNA, providing limited error correction. Despite such inherent error correction abilities, the use of error correction methods at the encoding stage is required to reliably retrieve information after many generations of a host organism.

Arita and Ohashi [4] developed an embedding algorithm which operates in pcDNA regions. The algorithm encodes binary data and was successfully tested *in vivo*. The main pitfall of this method is that it requires that the original DNA sequence be available at the decoder end in order to decode the embedded message.

One paper of significance was written by Heider and Barnekow [5], in which they proposed two versions of a data embedding algorithm, entitled “DNA-Crypt”. The ncDNA version of the DNA-Crypt algorithm is a trivial mapping of bits to bases. The authors also proposed a pcDNA version of their algorithm, and went on to test their proposal *in vivo*[14]. It was suggested that Hamming code be used in conjunction with DNA-Crypt to increase robustness under mutations, although note that error correction can actually be applied on any DNA data embedding method.

The use of repetition coding as an explicit DNA data embedding method was first proposed by Yachie *et al*[6]. The premise behind their algorithm is that errors may be corrected by embedding redundant copies of information throughout an organism’s genome. The authors performed *in vivo* embedding of binary data in multiple ncDNA regions. Also included was an *in silico* analysis of their method, showing the data recovery rate for a varying mutation rate. This work was expanded upon by Haughton and Balado [7].

The first paper to discuss performance analysis of data embedding algorithms and propose performance bounds was by Balado [15]. The achievable rate for both ncDNA and pcDNA under substitution mutations when codons are uniformly distributed was presented. Further bounds were proposed by Balado and Haughton in [16]. These are upper bounds on the possible embedding rate (bits per DNA component) that an algorithm can attain. Therefore we will compare the performance of the BioCode methods to these bounds.

For more information on DNA watermarking the reader is referred to the recent review by Heider and Barnekow [17].

### Notation and framework

In this section we introduce the notation necessary for explaining the BioCode algorithms. We also present the framework used and a summary of basic biological facts that will be needed to explain the algorithms. Sets will be represented by calligraphic letters, for instance S. The cardinality of a set, or the number of elements it contains, is denoted as |S|. Elements of sets are represented by lower case letters, such as v∈S. Vectors of elements are represented by bold letters, for instance **v**= [*v*_1_,*v*_2_,⋯,*v*_*k*_].

Inherently, DNA is a linear digital storage medium whose building blocks are four nucleotide bases, denoted in set notation by X≜{A,C,T,G}. These bases belong to two chemically distinct groups, purines R≜{A,G} and pyrimidines Y≜{T,C}. We will represent a DNA strand comprising *n* bases by a vector **x**= [*x*_1_,*x*_2_,⋯,*x*_*n*_], with $x_{i}\in X$. A dinucleotide DNA sequence is represented by a two-element vector **d**= [*x*_1_,*x*_2_]. The DNA molecule actually consists of two antiparallel strands, and either of the two strands completely defines the other by means of the so-called Watson-Crick base pairings A– T and G– C. This fact is of importance for the BioCode ncDNA method, as we will see later.

**Figure 1:**
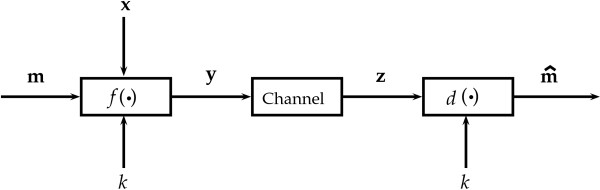
**Typical communications channel model.** An embedding function *f* (·) encodes a message **m** in a DNA sequence to produce **y**. If necessary this is done so with a host DNA sequence **x** and key *k*. **y** is transmitted through a channel to produce **z**, which is decoded using *d* (·).

The DNA data embedding problem may be modelled in terms of the communications channel shown in Figure 1. The purpose of DNA data embedding is to encode a message **m**= [*m*_1_,*m*_2_,⋯,*m*_*l*_], with $m_{i}\in ℳ≜\{0,1\}$, within a host DNA strand **x**. This is achieved using a function *f*(·,·,·), which represents a DNA data embedding algorithm. Its output is an encoded DNA strand **y**=*f*(**m**,**x**,*k*), where *k* is a secret key. Since organisms are subject to mutations, any information encoded in their genomes is equally so. This is reflected by **y** undergoing a probabilistic “mutations channel”, possibly accumulating errors, to give a mutated DNA strand **z**. At the decoder a function *d*(·,·) takes **z** in order to produce an estimate of the original message, **m**^′^=*d*(**z**,*k*). The embedding key *k* is a secret shared by the encoder and decoder to ensure that the encoded information is private. As we will see the embedding key may consist of a permutation of a basic translation table, but it may also include a cryptographic key if desired.

For reasons that will become clear next, DNA data embedding algorithms which target protein-coding DNA manipulate codons, as opposed to individual bases. A codon is a group of three consecutive bases, which we will denote as $\hat{x}=\;[x_{1},x_{2},x_{3}]\in X^{3}$, with a vector of codons being for instance $\bar{x}=\;[{\hat{x}}_{1},\cdots \;,{\hat{x}}_{n}]$. Genes are simply pcDNA regions flanked by certain start and stop markers enclosing consecutive codons^a^ that can be translated into proteins by the genetic machinery. Every codon $\hat{x}$ uniquely translates to an amino acid $a=\text{aa}(\hat{x})$, where the *aa*(·) function translates a codon (or codon sequence) to an amino acid (or amino acid sequence). Using their standard abbreviations, the set of all possible amino acids is A≜{Ala, Arg, Asn, Asp, Cys, Gln, Glu, Gly, His, Ile, Leu, Lys, Met, Phe, Pro, Ser, Thr, Trp, Tyr, Val, *Stp* }. *Stp* is included for notational convenience, although it is not an amino acid but just a “translation stop” command. The sequential concatenation of amino acids in a gene produces a protein. The relationship between codons and amino acids, represented by *aa*(·), is given by the near-universal genetic code. This is a redundant relationship since $|X^{3}|=64$ but |A|=21. The set of synonymous codons which translate the same amino acid a∈A is denoted $S_{a}$. The superset of all codons is given by $S_{A}$, and each subset $S_{a}$ is composed of the codons which translate the same amino acid, $\forall a\in A|S_{a}\subset S_{A}$. This redundancy is also behind the different *codon bias* (or codon usage bias) exhibited by different organisms. Codon biases are characteristic frequencies of the appearance of codons associated with each amino acid. As we will see, this built-in redundancy of the genetic code lies at the foundations of all pcDNA algorithms, and therefore both the genetic code and codon bias are fundamental to these techniques.

Finally, note that taking into account the three bases in a codon and the two antiparallel strands in a DNA molecule, there are six different reading frames in which a DNA segment could be translated to proteins. A correct reading frame is determined by the presence of a start codon (the codon mapping to Met, and two codons mapping to Leu in eukaryotic organisms).

### Constraints of DNA data embedding

It is essential that any data embedding process does not harm the functionality of the host organism, that is to say, the information-carrying DNA strand **y** and the original **x** should be biologically equivalent. In order to develop reliable data embedding algorithms the constraints which enable robust encoding must be clear. This section outlines important biological constraints which should be placed upon DNA modifications. The BioCode algorithms described in the following section abide by all of these constraints. 

• **ncDNA constraint**: *no start codons* — A modified ncDNA region (in order to embed information) should not be mistaken as a pcDNA region by the genetic machinery. This implies that start codons^b^ should not appear in the modifications. To the best of our knowledge BioCode ncDNA is the only algorithm strictly observing this constraint, however another method does acknowledge it to some extent. This algorithm was used by the JCVI to encode data in the artificially engineered synthetic bacterium and is disclosed in a patent [18]. This method does not completely guarantee that start codons will not be created; instead, it is designed such that the probability of start codons appearing is low. Moreover, this low likelihood only applies to one of the six possible reading frames of DNA, whereas BioCode ncDNA enforces the constraint in all six frames.

• In any case it might still happen that a modified region which originally did not contain start codons may acquire them due to mutations accumulated over a number of generations. This is clearly a potentially unavoidable scenario for any method.

• **pcDNA constraints**: *primary structure preservation* — The primary structure, i.e. protein translation, of a gene may not be altered, in effect meaning that $\text{aa}(\bar{y})=\text{aa}(\bar{x})$. Algorithms are restricted to encoding information by replacing codons synonymously (that is to say, with codons which translate the same amino acid). This greatly reduces capacity and increases the complexity of pcDNA algorithms over ncDNA algorithms. *codon bias preservation* (*codon count preservation*)— The second constraint which must be considered concerns the distribution of codons in organisms, or codon bias. There is a growing body of research pointing towards the codon bias usage of pcDNA regions dictating the gene expression levels in both eukaryotic and prokaryotic organisms, in particular, the speed at which genes are translated into proteins [19,20]. Therefore it is desirable that the codon bias in a given pcDNA region be preserved when such a region is modified to embed data. This constraint may be especially important when encoding information extensively throughout an organism’s genome.

• The empirical distribution of codons in a pcDNA region is given by its codon bias, which is just a normalised codon count. Hence, in practice preserving a codon bias amounts to preserving a codon count. In other words, the *codon bias preservation* constraint implies that the histogram of the codons in a pcDNA region must remain unchanged after the embedding process.

• It should be noted that if the codon composition for a particular amino acid does not vary, i.e. the same codon translates a single amino acid every time in a pcDNA region, then any algorithm operating under this constraint cannot encode information using those codons. In practice we have not observed this extreme case and while codon compositions do not appear with equal frequency, they are sufficiently distributed to achieve high embedding rates.

The codon bias preservation constraint has been acknowledged, to some extent, in a DNA embedding algorithm created by Liss et al. [8]. This algorithm encodes information by first determining the frequency of each codon to be used for embedding. Codons are assigned to bit values in such a way as to mirror the bit frequencies of the message with the codon usage frequencies. It is a reasonable assumption to expect the binary message to be embedded, **m** to be approximately uniformly random as any data will appear so when compressed. Under the method by Liss et al., if we assume the binary message is uniformly random, and there is high variation in codon usage frequencies for an amino acid, the codon bias would not be preserved.

An even more stringent constraint for pcDNA embedding is the preservation of codon pairs. A recent study demonstrated that certain codon pairs were preferred in pcDNA regions, while others were avoided [21]. We have investigated this constraint when combined with the two constraints above and, for the genes used in this study, have determined that no information can be encoded when strictly enforced. In these genes there were no two amino acid pairs with differing codon compositions, meaning that no codon pairs could be swapped while maintaining the primary structure preservation constraint. Therefore this constraint will not be considered here. A further issue with this constraint is the preservation of codon pairs in different reading frames. If codon pairs in all reading frames were to be preserved, the DNA sequence could not be modified at all.

## Method

As we will see, both algorithms proposed in this paper operate under conditions which vary depending upon the message encoding progress, and which take into account the aforementioned constraints on DNA modification. Both algorithms face the problem of statically or dynamically mapping a given set of available symbols (bases or codons) to message bits, and vice versa. For clarity, this common encoding principle which we call *graduated mapping* will be introduced next, before the actual BioCode algorithms are presented.

### Graduated mapping

Given a set of available symbols S, which in general are bases or codons, it is possible to map all of it’s elements to the elements of a second set of binary strings ℳ. Obviously both sets must have identical cardinality, denoted by μ=|S|=|ℳ|. Let $l≜\lfloor \underset{2}{\log}\mu \rfloor$ denote the minimum length of any binary string in ℳ.

First, let us consider the simplest case, that is, when *l*= log2*μ*. In this case ℳ is composed of *μ* length-*l* binary strings, arranged in ascending order from zero to *μ*−1. The other case to consider is when *l*< log2*μ*. In this instance, to achieve a higher embedding rate, some of the binary strings in ℳ must be of length ⌊log2*μ*⌋+1 bits. The first 2^*l*^ values from S are assigned *l*-length binary strings, in ascending order from 0 to 2^*l*^−1. The remaining values from the range 2^*l*^+1 to *μ* are first duplicated with the *l*-length binary strings corresponding to the range 2^*l*+1^−*μ*+1 to 2^*l*^. The strings in the former range are concatenated with a “1”, while the strings in the latter are concatenated with a “0”.

#### Dynamic graduated mapping

We will see that a special situation is the requirement that each of the elements from S be used a specific amount of times due to biological constraints. If an element s∈S has been used as many times as permitted, then it will be removed from S, decreasing *μ* by one unit. Every such removal prompts a remapping of S⇔ℳ in a graduated fashion, whereby ℳ is completely recreated using the new value of *μ* and the mapping method just described in the paragraph above.

As an example of the method, suppose that S={a,b,c,d,e}, then it would have the following mapping S⇔ℳ={00,01,10,110,111}. Now, if during execution of the algorithm the element *d* is used as many times as permitted, S becomes S∖d and the set ℳ is remapped as ℳ={00,01,10,11}.

As we will see in the following section, the two BioCode algorithms exploit the basic concept of graduated mapping in their own unique ways. Notice that the actual permutations used in the mappings may be kept as a secret shared by encoder and decoder, thus implementing the aforementioned secret key that precludes decoding by unauthorised third parties.

### BioCode ncDNA

In this section we introduce BioCode ncDNA —a method to optimally embed information within ncDNA while observing the *no start codons* constraint. Firstly, observe that as |X|=4 it is possible to encode information by trivially assigning a two bit sequence to each base. This is the foundation of the ncDNA embedding algorithm DNA-Crypt by Heider and Barnekow [5], among others. However such a static mapping of bits to DNA symbols does not take into account the *no start codons* constraint discussed in the previous section. Using such a mapping it is possible that some particular messages will produce start codons in the information-carrying strand. One might think that simply avoiding messages which translate into start codons would bypass this problem. However, this is far from being a solution because there are three possible reading frames where the genetic machinery might find a start codon, plus three additional reading frames in the antiparallel complementary strand.

In order to address this issue BioCode ncDNA uses a variable symbol mapping that we describe next. For generality it is assumed that the host DNA belongs to a eukaryotic organism, for which the start codons are “ATG”, “CTG” and “TTG”, with the complementary codons on the opposite strand being “CAT”,“CAG” and “CAA”. Taking the first two bases of these triplets, the following set of special duplets is defined:

$$\begin{array}{ll} D≜\{\text{AT, CT, TT, CA}\} & \; \end{array}$$

These duplets indicate that the next encoded symbol in a DNA sequence is a special case since a start codon may be produced if the wrong symbol is encoded. Such a situation is avoided by constantly examining the trailing dinucleotide sequence, **d**=[*y*_*i*−2_,*y*_*i*−1_], where *i* represents the position of encoding within the information-carrying DNA sequence **y**. If the concatenation of the previous two bases **d** with the current base *y*_*i*_ has the potential to create a start codon (that is, if d∈D), then the algorithm restricts the choice of *y*_*i*_ to a subset of bases $S_{d}$ such that no start codon can be produced. Otherwise *y*_*i*_ can be freely chosen from X. In order to reflect these conditions, a graduated mapping from the subset $S_{d}$ to message bits is used to encode the symbol *y*_*i*_. Note that the graduated mapping is different for different values of **d**, but static for any given **d**.

**Figure 2:**
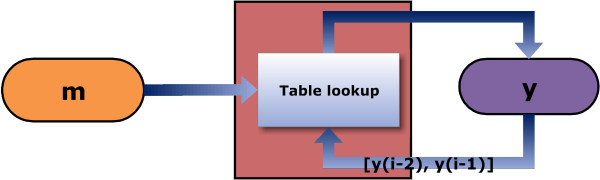
**A schematic of the BioCode ncDNA algorithm.** The input message **m**, in conjunction with the trailing dinucleotide sequence [*y*_*i*−2_,*y*_*i*−1_] is used to perform a lookup of Table 1.

A schematic of the algorithm is shown in Figure 2. The encoded DNA sequence **y** is constructed by reading the binary message **m** and at each point examining the previously encoded dinucleotide **d**. A lookup of Table 1 is performed using **d** and the next bit(s) to be encoded *m*, from the message vector **m**. The base $y\in S_{d}$ is selected for encoding using $m\in {ℳ}_{d}$. This mapping is performed by locating *m* in the set ${ℳ}_{d}$ and choosing the base *y* from $S_{d}$ at the corresponding position.

**Table 1:** BioCode ncDNA

**d**	**AT**	**CT**	**TT**	**CA**	$X^{2}\setminus D$
$\left|S_{d}\right|$	3	3	3	1	4
$S_{d}$	A	A	A	C	A
	T	T	T		T
	C	C	C		C
					G
	*↓*			Decode	
	Encode			*↑*	
${ℳ}_{d}$	0	0	0		00
	10	10	10		01
	11	11	11		10
					11

Given the dinucleotide sequence **d** the next message base to be encoded is one belonging to the set $S_{d}$. Each bit message found in ${ℳ}_{d}$ corresponds to a base in $S_{d}$.

BioCode ncDNA guarantees that no start codon can be created in all reading frames in both sense and anti-sense directions. The algorithm can be easily modified in such a way as to prevent any other codon of choice from appearing. Decoding an embedded message is simply the reverse process of encoding, with one additional improvement. Since it is not possible for start codons to appear intentionally, if they do arise due to mutations it is possible to detect the corresponding message errors —and even in some cases to correct them.

### Binary Codon equivalency

Before introducing BioCode pcDNA –a method to near optimally embed information within pcDNA while observing the *primary structure preservation* and *codon count preservation* constraints— we will briefly describe a pcDNA data embedding algorithm previously proposed by us, called Binary Codon Equivalency (BCE) [22]. BCE can be seen as a particular instance of BioCode pcDNA when only the *primary structure preservation* constraint is obeyed —but not the *codon count preservation* constraint. Central to BCE is a lookup table containing graduated mappings of codons to bit strings. Table 2 explicitly shows this mapping, with part **(a)** showing the genetic code and part **(b)** giving the translated bit sequences. It should be noted that this mapping has been refined since BCE was originally disclosed in [22], in order to achieve a higher embedding rate.

**Table 2:** Binary to Codon translation table

**(a)**																					
***a***	**Ala**	**Cys**	**Asp**	**Glu**	**Phe**	**Gly**	**His**	**Ile**	**Lys**	**Leu**	**Met**	**Asn**	**Pro**	**Gln**	**Arg**	**Ser**	**Thr**	**Val**	**Trp**	***Stp***	**Tyr**
$\left|S_{a}\right|$	4	2	2	2	2	4	2	3	2	6	1	4	2	2	6	6	4	4	1	3	2
	GCA	TGC	GAC	GAA	TTC	GGA	CAC	ATA	AAA	CTA	ATG	AAC	CCA	CAA	AGA	AGC	ACA	GTA	TGG	TAA	TAC
	GCC	TGT	GAT	GAG	TTT	GGC	CAT	ATC	AAG	CTC		AAT	CCC	CAG	AGG	AGT	ACC	GTC		TAG	TAT
$S_{a}$	GCG					GGG		ATT		CTG		CCG			CGA	TCA	ACG	GTG		TGA	
	GCT					GGT				CTT		CCT			CGC	TCC	ACT	GTT			
										TTA					CGG	TCG					
										TTG					CGT	TCT					
											*↑* Decode										
									Encode *↓*												
	**(b)**																					
	00	0	0	0	0	00	0	0	0	00	-	00	0	0	00	00	00	00	-	0	0	
	01	1	1	1	1	01	1	10	1	01		01	1	1	01	01	01	01		10	1	
${ℳ}_{a}$	10					10		11		100		10			100	100	10	10		11		
	11					11				101		11			101	101	11	11				
										110					110	110						
										111					111	111						

**(a)** Codon to amino acid translation table (genetic code). Underlined are codons which double as start codons. **(b)** Available binary strings for message encoding. There is a one to one mapping of each binary string in ${ℳ}_{a}$ to a codon in $S_{a}$, given by the table entries in the same positions.

BCE executes as follows: it initiates by translating the sequence of codons, $\bar{x}=[{\hat{x}}_{1},{\hat{x}}_{2},\cdots \;,{\hat{x}}_{n}]$ into its corresponding amino acid sequence $a=\text{aa}(\bar{x})=[a_{1},a_{2},\cdots \;,a_{n}]$ (primary structure). The encoded sequence, $\bar{y}$ is then constructed by traversing **a** and choosing for each index *i* a message-dependent codon ${\hat{y}}_{i}$ such that $\text{aa}({\hat{y}}_{i})=a_{i}$. A lookup of Table 2 is performed to find the bit sequence matching the current message bit(s) **m** in ${ℳ}_{a_{i}}$. The codon ${\hat{y}}_{i}\in S_{a_{i}}$ is selected corresponding to the position of that match.

### BioCode pcDNA

The BioCode pcDNA algorithm preserves in $\bar{y}$ not only the primary structure of the original host sequence $\bar{x}$ —as BCE does already— but also its codon count. These two objectives are simultaneously achieved by means of a dynamic adaptation of the strategy followed by BCE. We have just seen that in BCE the cardinality of the codon set $S_{a_{i}}$ corresponding to each amino acid *a*_*i*_ is constant for all *i*=1,2,⋯,*n*, which allows the use of a static lookup table throughout the embedding process. However the additional constraint observed by BioCode pcDNA requires the cardinality of $S_{a}$ to be varied during the embedding process.

**Figure 3:**
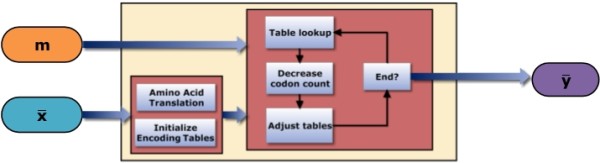
**A schematic of the BioCode pcDNA algorithm.** The message **m** and host DNA sequence $\bar{x}$ are inputs to the algorithm. The encoded sequence $\bar{y}$ is output, guaranteeing that the *codon bias preservation* and the *primary structure preservation* constraints are adhered to.

The following is a step by step procedure of the algorithms’ operation made with reference to Figure 3. 

• **Amino Acid Translation** — As in BCE, the vector of codons, $\bar{x}$ is converted into a vector of amino acids; $a=\text{aa}(\bar{x})$.

• **Initialize Encoding Tables** — Next, for every amino acid, all possible codon types in $\bar{x}$ which translate that amino acid must be found. Given $S_{c}$ is the set of *k* codons which translate a single amino acid, $S_{c}$ will only contain the codon types which appear in $\bar{x}$. If all *k* possible codon compositions are found in $\bar{x}$, then $S_{c}$ will contain all *k* codons. For example, given the amino acid Glycine we have the corresponding set $S_{g}$. Four codons translate this amino acid which would normally yield $S_{g}≜\{\text{GGA, GGC, GGG, GGT}\}$. However if the codon GGT does not appear in $\bar{x}$ and all other codons do, then the set will consists of $S_{g}≜\{\text{GGA, GGC, GGG,}\}$. This process of inserting all the codon types into their component amino acid sets continues until all the unique codons in $\bar{x}$ have been classified. For each amino acid set, a set identical in size is created to contain the corresponding bit mappings. Given $S_{c}$, a corresponding set ${ℳ}_{c}$ is populated using the cardinality μ=|C| and the graduated method described in the previous section. There is then a mapping of $S_{c}\mapsto {ℳ}_{c}$. $S_{c}$ is contained within a superset of codon sets, $S_{c}\in S_{A}$. If the full set of 64 codons are identified in the pcDNA region then the entire amino acid set $S_{A}$ and corresponding bit mappings ${ℳ}_{A}$ would be identical to Tables 2**(a)** and **(b)**. Once $S_{A}$ and ${ℳ}_{A}$ have been initialized for each amino acid, they may be queried to determine the available codons and possible bit sequences that can be encoded. Continuing the example above for G≜{GGA, GGC, GGG}, the possible bit mappings for G would be ${ℳ}_{g}≜\{0,10,11\}$.

• A codon count vector **c** is then created, which contains the number of times that each codon appears in a pcDNA region. This, along with $S_{A}$ and ${ℳ}_{A}$ will be modified as the algorithm progresses.

• **Table Lookup** — Construction of $\bar{y}$ begins by examining the first amino acid *a*_1_ and the first 3 bits in the message sequence, [*m*_1_,*m*_2_,*m*_3_]. If amino acid *a*_1_ is represented by the codon set $S_{a_{1}}$ (all codons in $S_{a_{1}}$ translate *a*_1_), then the available bit sequences are given by ${ℳ}_{a_{1}}$. The bit sequence matching the current input is searched for in ${ℳ}_{a_{1}}$, if $\{m_{1},m_{2},m_{3}\}\notin {ℳ}_{a_{1}}$, then {*m*_1_,*m*_2_} is located, if $\{m_{1},m_{2}\}\notin {ℳ}_{a_{1}}$ then {*m*_1_} is located. The position at which the matching bit sequence is located corresponds to the codon to be selected for embedding from $S_{a_{1}}$. That is to say, if the *k*-th element in ${ℳ}_{a_{1}}$ is identical to the current input, then the *k*-th codon of the same amino acid from $S_{a_{1}}$ is used for encoding.

• **Decrease Codon Count** — Once the codon $\hat{y}$ has been used for encoding, the count for that codon in **c** is decremented by one.

• **Adjust Tables** — If the count for codon $\hat{y}$ reaches zero, then codon $\hat{y}$ is removed from $S_{A}$. In other words, if a codon has been used as many times as it appeared in the original sequence then that codon can no longer be used for embedding because the budget for that codon has been exhausted. The removal of $\hat{y}$ from $S_{A}$ also prompts a remapping of $S_{\text{aa}(\hat{y})}\mapsto {ℳ}_{\text{aa}(\hat{y})}$ in a graduated fashion.

• **End** — The algorithm loops back to the **Table Lookup** step, continuing its iteration through **a** to produce $\bar{y}$, until the end of **m** or $\bar{x}$ has been reached.

Decoding is the reverse procedure of embedding. Instead of performing a lookup using the message vector, a lookup is performed using codons to retrieve the message vector. All of the tables created for encoding must also be created at the decoder and are modified during execution in the same way. An example of BioCode pcDNA encoding with step by step procedure is demonstrated in an Additional file 1. This includes codon and amino acid statics for $\bar{x}$ and $\bar{y}$.

### Information embedding rate of the BioCode Algorithms

In this section we analyse the information embedding rate of the BioCode algorithms in message bits/base or message bits/codon. In order to do so we will first discuss the embedding rate of the graduated mapping method, which assigns symbols (bases or codons) to bits in both BioCode methods. For simplicity we will assume that the message bits are uniformly distributed at random.

The graduated mapping method can achieve a near-optimal rate in terms of bits/symbol (that is, in bits/base or in bits/codon). Its minimum embedding rate *R*_*↓*_ for a given codebook size *μ* is:

(1) $$\begin{array}{ll} R_{↓}=\left\lfloor \underset{2}{\log}\mu \right\rfloor \text{bits/symbol} & \; \end{array}$$

The maximum embedding rate is simply *R*_*↑*_=*R*_*↓*_+1. Therefore the average embedding rate is

(2) $$\begin{array}{ll} \;R(\mu )=R_{↓}\;\times \;\left(\frac{2^{R_{↑}}-\mu }{2^{R_{↓}}}\right)+R_{↑}\;\times \;\left(\frac{2\mu }{2^{R_{↑}}}-1\right)\text{bits/symbol}\; & \; \end{array}$$

The equation above may be explained as the weighted average of the lower embedding rate, *R*_*↓*_, and the higher embedding rate, *R*_*↑*_, using as weights the probabilities of those rates being implemented by the encoder. The optimal achievable rate, independent of any method, is given by *R*= log2*μ*. There exists one method which attains this rate, called arithmetic encoding [3]. However arithmetic encoding presents error propagation issues at the decoder, which make it impossible to implement error correction effectively.

#### BioCode ncDNA

**Figure 4:**
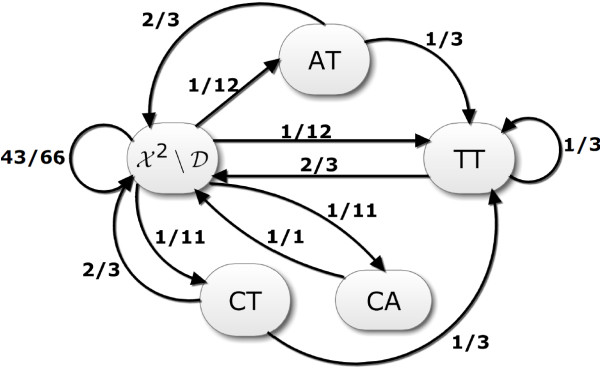
**Markov chain representing the probability of transition between trailing dinucleotide states.**$X^{2}\setminus D$ in this diagram represents all the dinucleotide sequences excluding those which may create start codons.

There are five states that the BioCode ncDNA encoder may be in, each of which is given by the trailing dinucleotide. These five states are “AT”, “CT”, “TT”, “CA” (i.e., the elements in D) and $X^{2}\setminus D$. In order to compute the average embedding rate of BioCode ncDNA we will obtain the steady state probability of the encoder being in each of the different states. The dynamic behaviour of this finite state machine may be modelled by means of the Markov chain shown in Figure 4. The state transition probabilities associated with this Markov chain, which are also given in the figure, can be obtained by examining the probabilities of using bit sequences given by Table 1. These transition probabilities can be used in turn to define the 5×5 transition probability matrix $T≜[\mathrm{Pr}(s_{\text{next}}|s_{\text{current}})]$, with $s_{\text{current}},s_{\text{next}}\in D\cup (X^{2}\setminus D)$. We wish now to obtain the steady state matrix $T^{\infty }=\underset{k\to \infty }{\lim}T^{k}$. In order to do this we first compute the diagonal matrix **d** containing the eigenvalues of **T**, and a matrix **P** whose columns contain the corresponding eigenvectors, such that **T**=**P**×**D**×**P**^−1^. With this decomposition we can write $T^{\infty }=\underset{k\to \infty }{\lim}P\times D^{k}\times P^{-1}$. As *k*→*∞*, **D**^*k*^ becomes an all-zero matrix, except for the top leftmost element becoming the unity. We can then take the any row vector of **T**^*∞*^ as steady state probability vector. The formula to compute the rate of BioCode ncDNA is given below, where *R*(·) is the rate function given by equation (2). The row elements of **T** are the marginal probabilities that the previous two bases are the dinucleotide corresponding to that row. These probabilities correspond to the Pr(**d**) part of the formula below.

(3) $$\begin{array}{ll} \;R_{\text{ncDNA}}=\underset{d\in D\cup (X^{2}\setminus D)}{\sum }\mathrm{Pr}(d)R(|S_{d}|)=1.7462\;\text{bits/base} & \; \end{array}$$

This embedding rate is not overly lower than the unconstrained rate of embedding of 2 bits/base. However this rate may only be attained when the message is long.

#### BioCode pcDNA

The embedding rate of BioCode pcDNA is more difficult to analyse due to the dynamic nature of the graduate mapping it relies upon. However it was shown in [16] that when the codon distribution is uniform and the host sequence is long the rate of the optimum DNA data embedding with codon bias preservation can be approximated by the rate of optimum DNA data embedding without this constraint. Therefore we will settle for approximating the BioCode pcDNA embedding rate by the BCE rate, assuming that the conditions above hold. The embedding rate of BCE is given by the equation below:

(4) $$\begin{array}{ll} R_{\text{BCE}}=\underset{a\in A}{\sum }\frac{\left|S_{a}\right|}{64}\times R(|S_{a}|)=1.75\;\text{bits/codon}, & \; \end{array}$$

where we have used expression (2). In order to see that this rate is near-optimum, observe that the maximum rate —independent of any method— may be calculated using the same formula above by replacing $R(|S_{a}|)$ with $\underset{2}{\log}|S_{a}|$. This gives a rate of 1.7819 bits/codon, which is only 3% higher than the BCE rate.

### Mutation channel model

In the following we will discuss the mutations model used to evaluate the performance of the BioCode methods. It must be emphasised that most previous authors proposing DNA data embedding did not provide decoding performance analyses of their algorithms, either by means of analyses or by means of *in silico* Monte Carlo simulations. An exception would be the work of Yachie et al. However such analyses are fundamental for understanding the expected performance of DNA data embedding methods when used in *in vivo* environments.

Performance analyses are important because the information embedded in the genome of an organism may contain errors caused by mutations accumulated after successive generations of the organism. That is, as shown in Figure 1, due to the effect of a “mutations channel” the information-carrying DNA sequence (**y**) may be transformed into a “noisy” version of it (**z**) before reaching the decoding stage. These errors may impair or degrade the decoding of the embedded information, and hence it is fundamental to analyse the algorithms’ performance under mutations.

Following the communications simile, the mutations channel causing the errors can be characterised using a probabilistic model. The model used in our analysis will only consider base substitution mutations, which are the most prevalent mutations in the DNA of bacteria. In particular such mutations are the overwhelming majority in pcDNA regions [23]. These mutations randomly replace one base with an alternate base at different loci of a genome, and therefore can be modelled by means of a 4×4 transition probability matrix Π≜[Pr(z|y)], where z,y∈X. As a simplification we will also consider that base substitution mutations happen independently at different loci. In reality it may happen that dependent mutations occur, for instance affecting a number of consecutive bases. However such dependencies can be easily broken by any information embedding method by means of a pseudo-random interleaver shared by encoder and decoder.

The simplest —and one of the most commonly used— models of base substitution mutation is the Jukes-Cantor model of molecular evolution, which assumes that Pr(*z*|*y*)=*q*/3 for *z*≠*y* and Pr(*y*|*y*)=1−*q*. Therefore *q*=Pr(*z*≠*y*|*y*) is the base substitution mutation rate. However the mutation model used in our *in silico* analysis is the more realistic Kimura model of [24], whose transition probability matrix is

(5) $$\Pi =\left[\begin{array}{cccc} A & C & T & G\\ 1-q & \frac{\gamma }{3}q & \frac{\gamma }{3}q & (1-\frac{2\gamma }{3})q\\ \frac{\gamma }{3}q & 1-q & (1-\frac{2\gamma }{3})q & \frac{\gamma }{3}q\\ \frac{\gamma }{3}q & (1-\frac{2\gamma }{3})q & 1-q & \frac{\gamma }{3}q\\ (1-\frac{2\gamma }{3})q & \frac{\gamma }{3}q & \frac{\gamma }{3}q & 1-qG \end{array}\right]\;\begin{array}{c} A\\ C\\ T\\ G \end{array}$$

This model can reflect the higher probability of base transitions (mutations among purines or among pyrimidines) over base transversions (mutations between purines and pyrimidines) by setting *γ*<1. The *γ* parameter is a function of the ratio of transitions to transversions *ε*, and it is obtained from it as *γ*=3/(2(*ε*+1)). This model becomes the less realistic Jukes-Cantor model when *γ*=1. For a more in-depth explanation the reader is directed to [7].

Since mutation events occur from parent to child it is natural to model the mutation channel for the number of generations *p* elapsed between **y** and **z**. Assuming that *Π* gives the transition probability matrix for one generation, the model for *p* generations is easily found as *Π*^*p*^. We denote this straightforward extension as a “cascaded mutations model”.

At most, a mutation model can have nine parameters if it the property of time reversibility is to hold. The Kimura model is used in place of models with greater numbers of parameters because of the statistical problem of overfitting. If a mutation model has several parameters, some of which cannot be accurately estimated, the results obtained after many generations will be distorted. Reliable estimates of *q* and *γ* are available and therefore *Π*^*p*^ can be accurately approximated. The Kimura model has been proven accurate in predicting the capacity of a DNA sequence when compared with a 12 parameter model [25].

### Message bitframe resynchronisation

While performance will only be evaluated under the base substitution mutation channel just described, base errors may also occasionally confuse the decoder into inserting or removing message bits. If this happens the message bitframe common to encoder and decoder can become desynchronised, that is, the same index in **m** and **m**^′^ may no longer refer to the same message bit. We must stress that this issue not confined to BioCode, but common to all existing pcDNA data embedding algorithms. Therefore, the message bitframe must be resynchronised at the decoder, as otherwise the situation above may occasionally lead to a high message bit error.

We will employ two resynchronisation methods in order to deal with bitframe desynchronisation errors: marker codes and watermark codes. These strategies could actually be applied to resynchronise after insertion and deletion mutations on the level of DNA, which are not considered in this paper. Since they are applied on the bit level, not quaternary, the methods would lack channel information and as such can not decode optimally. Incorporating these methods fully for the DNA case is no trivial task because the embedding rate per base is not constant when operating under the restriction highlighted in this paper.

#### Marker codes

Marker codes were originally proposed by Sellers [26] in 1962, however they were not referred to as marker codes until much later [27]. These codes place a pilot signal at regular intervals in the binary message. The decoder expects the pilot signal to be located at specific points and if not found corrective action is taken. Suppose the pilot signal “001” is received by the decoder as “010”, it would infer that a deletion has occurred in the block preceding the pilot. The decoder resynchronises the remainder of the message by inserting a bit in the middle of the erroneous block. Marker codes, in the original proposal, are capable of correcting one desynchronisation error per block. They are not, however, designed to correct the block in which the error occurred.

#### Watermark codes

Watermark codes are a recently proposed resynchronisation method by Davey and MacKay that have been shown to achieve a high encoding rate [27]. Despite their name, they are not related to DNA watermarking, but may be applied here to correct bit insertions and deletions. The application a watermark code is as follows: firstly a “watermark” vector **w** is created which, for the purpose of our simulations was a uniformly random binary vector agreed upon by the encoder and decoder. The *sparse*(·) function inserts zeros evenly throughout the input binary vector with the position of insertions known to both encoder and decoder. The message vector, **m** is sparsified and added modulo 2 to the watermarked vector, $\tilde{m}=\text{sparse}(m)+w$, which is then embedded in a DNA sequence.

The next step differs in our implementation over Davey and MacKay’s. Under their method, after being transmitted across a channel, the received vector ${\tilde{m}}^{\prime }$ is processed by the Forward Backward algorithm to correct insertions and deletions [27]. However under our method, after the DNA sequence has been decoded, possibly accumulating errors, the watermark decoder processes ${\tilde{m}}^{\prime }$ by aligning it with **w**. This is done in a similar manner to the alignment process of the Needleman-Wunsch algorithm, however here the edit distance is used. One important factor must be incorporated into the alignment scoring; it is impossible for desynchronisation errors to occur in **w**. Differences between ${\tilde{m}}^{\prime }$ and **w**, other than desynchronisation errors, are a result of encoding or mutations, which the decoder is unable to distinguish between. Therefore, when resolving differences caused by flips between ${\tilde{m}}^{\prime }$ and **w**, the values in ${\tilde{m}}^{\prime }$ are stored as the alignment.

The Forward Backward algorithm allows for the computation of probabilities which may then be passed to a substitution error correction decoder. The method we employ here does not incorporate the channel transition probabilities in the realignment process and because of this, is not as accurate as the Davey and MacKay’s algorithm. However, our method is greatly simplified and less computationally complex.

## Results and discussion

In this section we describe the performance measures used to evaluate the BioCode algorithms. These evaluations are performed by means of Monte Carlo simulations, which implement the cascaded Kimura model as the mutations channel.

### Performance measures

First of all we must establish relevant and objective criterion for evaluation. A very important figure of merit is the “raw” probability of bit error at the decoder (*P*_*b*_), which is the probability that a bit will be incorrectly decoded after transmission across the mutations channel. By “raw” we mean without error correction coding (ECC): observe that ECC can be applied to any DNA data embedding method in order to enhance performance, but it is the baseline raw probability of bit error that determines the effectiveness of such additional strategies.

When evaluating *P*_*b*_ the Hamming distance is used as a metric for measuring the distance between two binary strings. The Hamming distance is the number of different same index symbols between two vectors, and it can be written using the Kronecker’s delta function *δ*(·,·) as $d_{H}(m,m^{\prime })=\overset{l-1}{\underset{i=0}{\sum }}\delta (m_{i},m_{i}^{\prime })$, where *l* is the message length in bits. Using this distance the average probability of bit error at the decoder is just

(6) $$\begin{array}{ll} P_{b}^{H}=\frac{1}{l}d_{H}(m,m^{\prime })=\frac{1}{l}\overset{l-1}{\underset{i=0}{\sum }}\delta (m_{i},m_{i}^{\prime }). & \; \end{array}$$

If no bitframe resynchronisation is applied, it can happen that $P_{b}^{H}$ is disproportionately high, even though only a few bits might have been inserted or deleted by the mutations channel.

We will also evaluate the potential performance of the BioCode algorithms when using optimal error correction coding. This will be done so by means of the mutual information between the message at the encoder (**m**) and at the decoder (**m**^′^). The mutual information is an information-theoretical measure which indicates the maximum amount of information that a communications system can send through a channel. Since the mutual information must always be below the Shannon capacity of the channel, we will be able to compare our results with the theoretical capacity limits for DNA data embedding computed in previous works [25]. In our simulations the mutual information was empirically obtained by comparing one bit of the original message, *m*, with its estimate after decoding, *m*^′^, as follows:

(7) $$\begin{array}{ll} \;I(M;M^{\prime })=\underset{m^{\prime }\in ℳ}{\sum }\underset{m\in ℳ}{\sum }\text{Pr}(m,m^{\prime })\underset{2}{\log}\left(\frac{\text{Pr}(m,m^{\prime })}{\text{Pr}(m)\text{Pr}(m^{\prime })}\right), & \; \end{array}$$

where Pr(·) are empirical estimates of these probabilities computed from the Monte Carlo experiments. We note that *I*(*M*;*M*^′^) must be scaled from bits/bit to bits/base (for ncDNA methods) or bits/codon (for pcDNA methods).

### Monte Carlo simulations

The parameters used in the cascaded Kimura model are *q*=10^−8^ and *γ*=0.1, which are values used in prior work [7] and are based on realistic estimates obtained in [28]. The results for BioCode ncDNA were obtained using messages of length 10,000 bits. For BioCode pcDNA the message length varied depending on codon composition and host sequence length. All the graphs compare either the mutual information or probability of bit error ($P_{b}^{H}$) against the number of generations an encoded sequence has been transmitted along.

**Figure 5:**
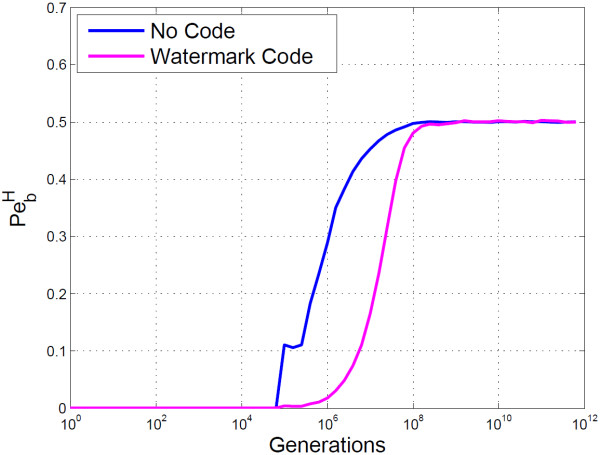
**Empirical results of BioCode ncDNA.** Shown is the probability of bit error using the Hamming distance ($P_{b}^{H}$), for BioCode ncDNA (blue). Also shown is $P_{b}^{H}$ for BioCode ncDNA, first encoded with a watermark code (purple).

The BioCode ncDNA $P_{b}^{H}$ graph shown in Figure 5, clearly demonstrates that information can be correctly retrieved up to 10^4^ generations of a host organism under the cascaded Kimura model. Also shown in the graph is BioCode ncDNA’s performance when the message is first encoded with a watermark code. This yields a significant improvement, allowing for errorless information retrieval up to 10^5^ generations. Marker codes, in this case, did not decrease $P_{b}^{H}$. If desynchronisation errors are rare and bit flips common, a marker code may itself cause desynchronisation errors due to the misinterpretation of error types. Also, marker codes cannot correct the block in which the desynchronisation error occurred, only resynchronise the remainder of the message. Thus if blocks are large relative to the entire message length, $P_{b}^{H}$ may be high.

**Figure 6:**
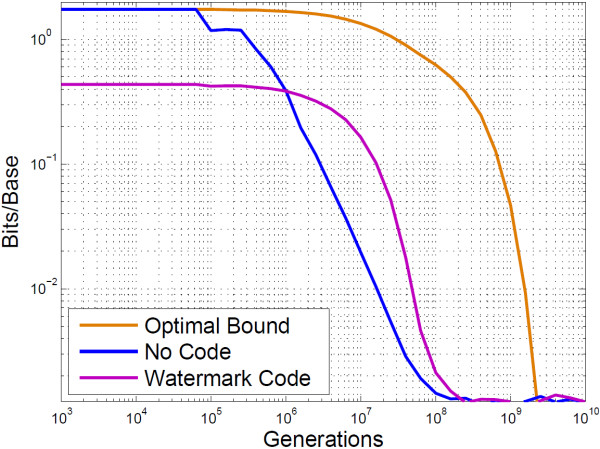
**Empirical results of BioCode ncDNA.** This is a log-log plot of the mutual information content of BioCode ncDNA compared to an optimal bound. Also shown is BioCode ncDNA encoded with the watermark code. Information content is given in bits/base.

With error correction against bit flips the $P_{b}^{H}$, for BioCode with a watermark code, could be further reduced for generations beyond 10^7^, at the expense of decreasing the embedding rate. Similarly the mutual information plot shows that 1.75 bits/base may be retrieved up to just beyond 10,000 generations. Figure 6 compares the mutual information of BioCode ncDNA against an optimal bound computed using the Blahut-Arimoto algorithm (Computation provided in [29]), and shows that the algorithm is optimal up to 10^5^ generations.

For the empirical analysis of BioCode pcDNA three different pcDNA regions were selected for embedding, two of which were used in prior works. The “ftsZ” region ^c^ in the *B. subtilis* genome was used for *in vivo* data embedding with Arita and Ohashi’s algorithm [4]. The “ypt7” region ^d^, from a species of yeast known as *S. cerevisiae*, was used for *in silico* data embedding with the DNA-Crypt algorithm [5]. The other region used, “pSD1_197” is a plasmid gene of a bacteria belonging to the Shigella genus ^e^, selected for its differing codon composition and larger sequence length.

**Figure 7:**
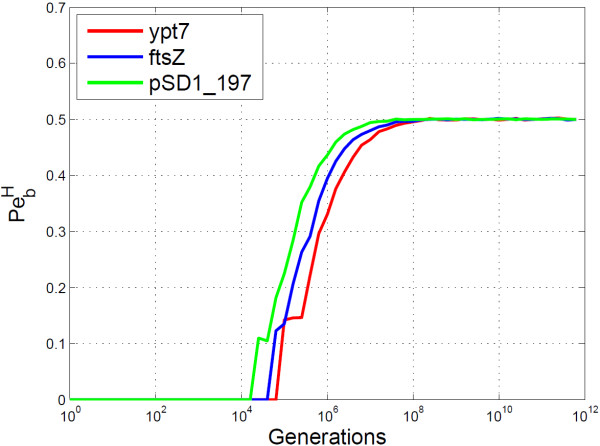
**Empirical analysis of BioCode-pcDNA for different genes.** Shown is the probability of bit error using the Hamming distance ($P_{b}^{H}$). BioCode pcDNA was used for encoding the data. Two of the genes have been used for encoding data in prior works [4,5].

**Figure 8:**
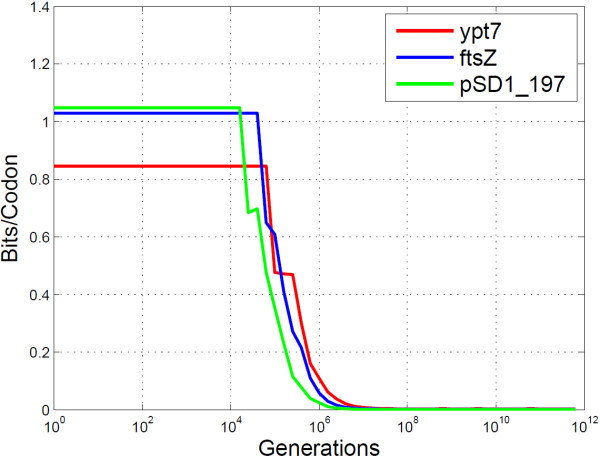
**Empirical analysis of BioCode-pcDNA for different genes.** The mutual information content for three genes encoded with BioCode pcDNA is shown. It is given in bits/codon.

The $P_{b}^{H}$ analyses of BioCode pcDNA on the three genes mentioned above, shown in Figure 7, clearly shows errorless data retrieval up to 10^4^ generations. However, the embedding rate varied significantly, as shown by Figure 8, with the rate of the“ypt7” gene (0.845 bits/codon) being considerably lower than the other two (“ftsZ”: 1.03 bits.codon and “pSD1_197”: 1.05 bits/codon). An interesting phenomenon of BioCode is responsible for this difference, namely that as sequence length increases so to does embedding rate. The “ypt7” gene is only 624 bases long, while the “ftsZ” and “pSD1_197 ” genes are 1158 bases and 3309 bases long respectively. In effect, it is more efficient at data storage for greater sequence lengths due to a greater number of possible combinations of codons and positions to choose from.

**Figure 9:**
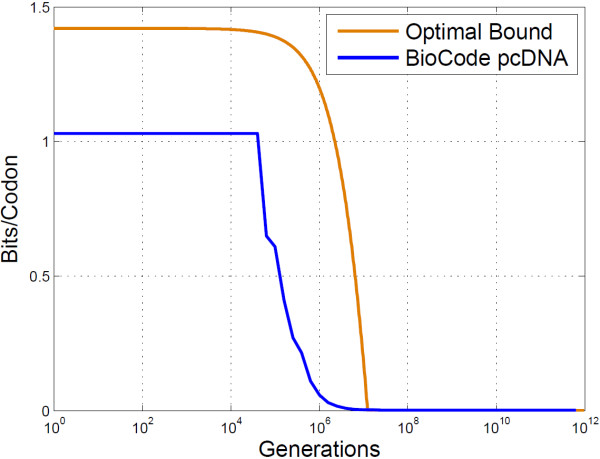
**BioCode pcDNA versus optimal bound.** The mutual information content for BioCode pcDNA and the optimal bound. The gene used for encoding and in computing the bound was the “ftsZ” gene.

A theoretical method for computing the optimal embedding rate when observing the *primary structure preservation* and *codon count preservation* constraints is described in [16]. This bound can be determined by means of a combinatorial analysis of the maximum number of ways codons in a gene may be rearranged while keeping the constraints. Figure 9 compares this optimal bound with BioCode pcDNA using the “ftsZ” gene.

**Figure 10:**
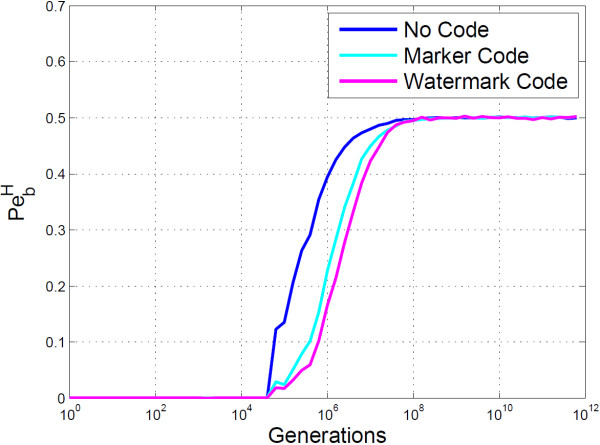
**Empirical analysis of BioCode-pcDNA using resynchronisation error correction.** Shown is the probability of bit error using the Hamming distance for BioCode pcDNA alone (blue), BioCode pcDNA using a marker code (light blue) and BioCode pcDNA using a watermark code (purple). The gene used was the “ftsZ” gene.

The remainder of the plots were obtained using the “ftsZ” gene for encoding. Figure 10 shows that when marker and watermark codes are used in conjunction with BioCode pcDNA they pose a considerable improvement. This is true despite not being capable of correcting flips in the message, which would account for the overwhelming majority of mutations. From this plot it is apparent that the watermark code reduces the $P_{b}^{H}$ more so than marker codes.

**Figure 11:**
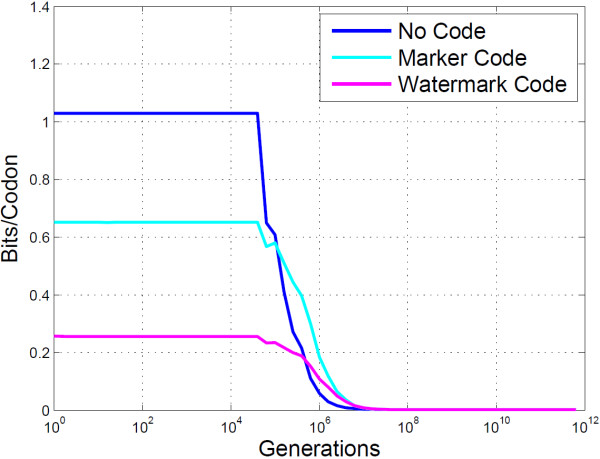
**Empirical analysis of BioCode-pcDNA using resynchronisation error correction.** This plot shows the mutual information content for BioCode pcDNA alone, with a marker code and with a watermark code.

**Figure 12:**
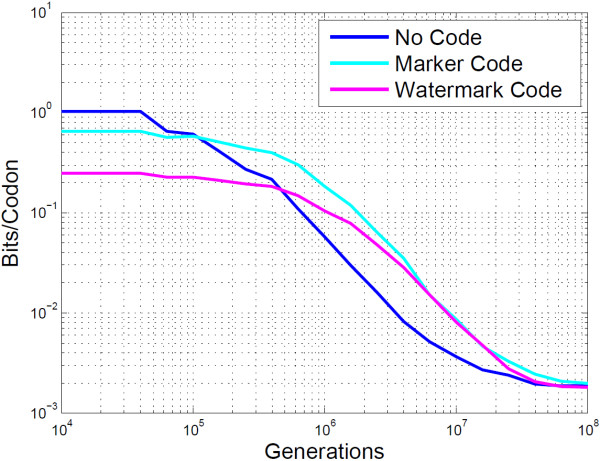
**Empirical analysis of BioCode-pcDNA using resynchronisation error correction.** This is a log-log plot of Figure 11 from 10^4^ to 10^8^ generations, showing the mutual information content for BioCode pcDNA alone, with a marker code and with a watermark code.

It is important to note the gradient of the plots, as they demonstrate that errors incurred from mutations are isolated and do not propagate. If this were not the case the $P_{b}^{H}$ would be greater between 10^4^ and 10^6^. Figure 11 compares the mutual information of the two error correction methods with no code. It clearly shows that the marker code outperforms the watermark code in terms of embedding rate. A more informative view highlighting this improvement is shown in Figure 12.

Finally, the last set of graphs compare BCE with algorithms proposed by other authors. Notice that the constraints under which the BioCode algorithms operate have never fully been incorporated into any previous embedding method. Therefore direct comparisons with other methods are not appropriate (although comparisons against theoretical bounds are still possible). However BCE, which may be seen as a particular instance of BioCode pcDNA, can actually be compared to other pcDNA data embedding algorithms. Heider and Barnekow’s DNA-Crypt [5] and Arita and Ohashi’s method [4] are compared to BCE. These methods only maintain the *primary structure preservation* constraint.

**Figure 13:**
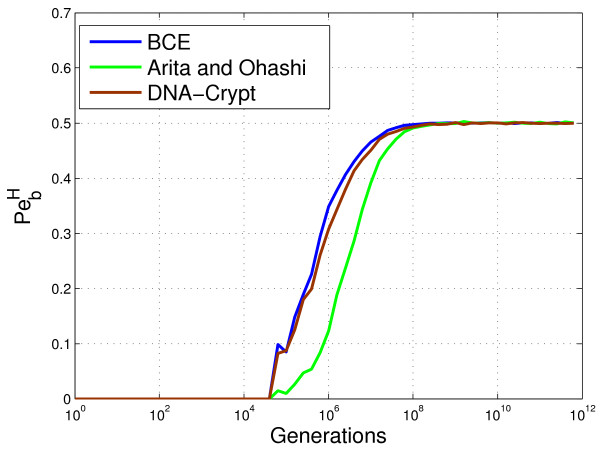
**Empirical analysis of BCE, Arita’s algorithm and DNA-Crypt.** This probability of bit error plot compares binary codon equivalency (BCE), Arita and Ohashi’s algorithm and DNA-Crypt. Arita and Ohashi’s algorithm requires that the original DNA sequence be available for decoding. BCE is a particular instance of BioCode pcDNA when the *codon bias preservation* constraint is not applied.

**Figure 14:**
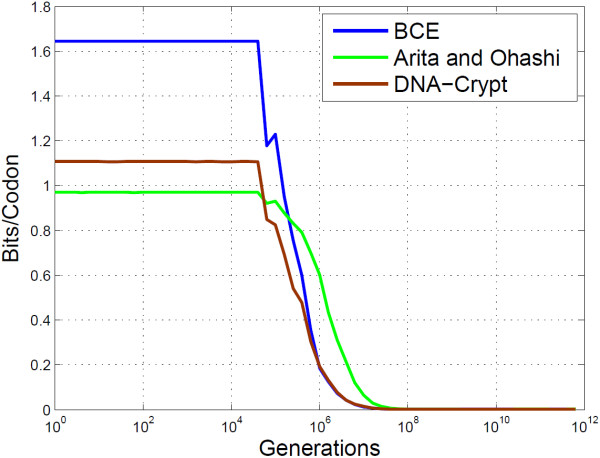
**Empirical analysis of BCE, Arita’s algorithm and DNA-Crypt.** This plot shows the mutual information content of BCE, Arita and Ohashi’s algorithm and DNA-Crypt.

BCE and DNA-Crypt perform near identically in terms of $P_{b}^{H}$ (see Figure 13), however there is a major gain in embedding rate when using BCE, as shown in Figure 14. Both BCE and DNA-Crypt do not require any side information at the decoder, however Arita and Ohashi’s algorithm requires the original DNA sequence to decode. Such knowledge, which is unrealistic in practice, increases the robustness when insertions and deletions are possible. Also, since the embedding rate is constant for codons which have at least one other synonymous codon, the effects of de-synchronisation errors are limited, as can be seen by the shape of the mutual information curve in Figure 14. Notice the similarity in shape shown to that of Figure 11 for marker and watermark codes.

## Conclusions

In this paper we have introduced the BioCode algorithms for embedding information in DNA. These novel methods are designed to be more biologically compatible than any previous DNA data embedding algorithms, fully adhering to strict constraints. Furthermore they lay the foundation for information storage in DNA in a way that is both efficient and robust, as we have shown by means of *in silico* Monte Carlo simulations. The BioCode pcDNA algorithm preserves codon statics making it difficult to infer that information has been embedded. This aspect, in addition to BioCode pcDNA’s near-optimum embedding rate, implies that BioCode pcDNA is a near-optimum first-order steganographic method. While DNA data embedding is currently in its infancy, it is likely that this field will grow considerably as technologies for synthesising and sequencing DNA become cheaper and faster. Therefore efficient data embedding techniques such as the BioCode algorithms can potentially find widespread applicability.

## Endnotes

^a^Possibly interspersed with noncoding regions (introns) in eukaryotic cells.^b^Codons which mark the start of a gene in pcDNA.^c^[GenBank:NC_000964.3 (1597832..1598980)]^d^[GenBank:NC_001145.3 (267174..267800)]^e^[GenBank:NC_007607.1 (170357..173665)]

## Competing interests

A patent has been filed for the BioCode algorithms in Ireland.Both authors declare no other competing interests.

## Authors’ contributions

DH BioCode algorithms conception, development and theoretical analysis, software development and simulations, tables and figures preparation, manuscript preparation (main author). FB theoretical limits, manuscript preparation (secondary author), coordination, research funds collection. Both authors read and approved the final manuscript.

## Supplementary Material

Additional file 1 This file contains an example of BioCode pcDNA encoding a message into a DNA sequence. Click here for file
