# Supplementary material for: BioCode: Two biologically compatible Algorithms for embedding data in non-coding and coding regions of DNA
Source: BMC Bioinformatics. 2013 Apr 9;14:121. doi: 10.1186/1471-2105-14-121 (PMC3698116; doi:10.1186/1471-2105-14-121)
Supplement: Additional file 1 — This file contains an example of BioCode pcDNA encoding a message into a DNA sequence. [file 1471-2105-14-121-S1.pdf]

An Example Embedding using BioCode pcDNA

Message to Embedded:

“BioCode preserves codon bias”

m — Text to Binary (7-bit ASCII) Translation:

10000101101001110111111000011110111111001001100101010000011100001110010110010111001111
00101111001011101101100101111001101000001100011110111111001001101111110111001000001100
01011010011100001111001 1

x̄ — Host Sequence:

ATGTCTTCTAGAAAAAAAAATATTTTGAAAGTAATCATCCTAGGTGACTCTGGTGTTGGGAAGACCTCCTTGATGCACCGTTATGTCAAT
GATAAGTATTCTCAACAGTATAAAGCAACAATTGGGGCTGACTTTTTAACAAAAGAGGTGACAGTTGACGGTGATAAAGTTGCCACCATG
CAAGTTTGGGATACTGCTGGACAGGAACGTTTCCAATCACTGGGTGTTGCTTTCTATAGAGGTGCAGATTGTTGCGTTTTGGTCTACGAT
GTGACCAATGCCAGTTCCTTTGAGAATATTAAGTCTTGGCGAGATGAATTTCTAGTGCATGCGAACGTAAACTCACCAGAAACATTTCCA
TTTGTTATACTGGGAAATAAAATTGATGCCGAAGAATCTAAAAAAATTGTATCAGAAAAGTCCGCTCAGGAGCTTGCTAAATCATTAGGC
GATATTCCTTTGTTTTTAACAAGTGCCAAAAACGCCATAAACGTTGATACCGCATTTGAAGAAATTGCAAGGAGTGCTTTACAACAGAAT
CAAGCTGATACAGAAGCCTTTGAAGATGACTATAATGATGCCATCAATATTCGCCTAGATGGAGAAAATAATTCTTGTAGCTGT

a — Amino Acid Translation of x̄:

MSSRKKNILKVIILGDSGVGKTSLMHRYVNDKYSQQYKATIGADFLTKEVTVDGDKVATMQVWDTAGQERFQSLGVAFYRGADCCVLVYD
VTNASSFENIKSWRDEFLVHANVNSPETFPFVILGNKIDAEESKKIVSEKSAQELAKSLGDIPLFLTSAKNAINVDTAFEEIARSALQQN
QADTEAFEDDYNDAINIRLDGENNSCSC

ȳ — Encoded Sequence:

ATGTCAAGCAGGAAAAAGAATATACTTAAAGTTATCATTTTGGGGGACAGTGGTGTTGGCAAGACTTCTCTAATGCATAGATATGTGAAC
GATAAATATTCACAACAATACAAAGCCACCATCGGAGCAGATTTTTTAACAAAGGAAGTTACAGTCGACGGTGATAAGGTAGCTACCATG
CAGGTATGGGATACAGCTGGTCAAGAACGCTTTCAGTCCTTGGGAGTTGCATTTTATAGAGGTGCTGACTGCTGTGTTCTGGTATATGAC
GTCACAAATGCGAGTTCTTTTGAGAACATTAAATCTTGGCGTGATGAGTTCCTGGTGCACGCAAATGTTAACTCTCCTGAGACCTTTCCA
TTCGTTATTCTAGGAAATAAAATAGATGCAGAAGAAAGTAAAAAAATCGTTTCCGAAAAATCAGCCCAGGAACTAGCTAAATCCTTGGGG
GATATTCCATTATTTTTGACCTCTGCCAAAAACGCCATTAATGTGGATACAGCCTTTGAAGAAATTGCTCGTTCTGCTTTACAACAAAAT
CAGGCTGATACAGAAGCCTTTGAAGATGATTATAATGATGCCATTAATATTCGATTAGATGGTGAAAATAATTCTTGTTCATGT

m̂ — Decoded Binary

10 00 01 0 1 1 0 10 0 11 10 11 11 10 0 00 11 11 01 1 11 11 00 1 00 1 10 0 1 0 1 01 0 0 0 0 01 1 10
0 00 1 1 10 0 1 0 11 0 01 0 11 1 1 00 11 1 1 00 1 0 11 11 0 0 10 1 1 10 11 0 11 00 1 0 11 11 0 0 11
01 00 0 0 0 1 10 00 11 1 1 0 11 11 1 1 0 01 0 0 1 1 0 11 1 1 1 1 0 1 11 0 0 1 0 0 00 0 1 10 0 0 1 0
1 10 1 0 0 1 1 1 0 0 0 0 1 1 1 1 0 0 1 1

Binary to Text Translation:

“BioCode preserves codon bias”

Figure 1: An example of a BioCode pcDNA encoding. First the message “BioCode preserves codon bias” is translated (including white spaces) into a binary message **m**. Then BioCode embeds **m** in a host sequence **x̄** to produce **ȳ**. The host sequence used in this instance is the ypt7 region ([GenBank:NC_001145.3 (267174..267800)]), which was also used by Heider et al. in their *in silico* analysis. Each unique colour in the **x̄** and **ȳ** sequences represents the translated amino acid for that codon. The amino acid sequence **a** is translated from **x̄**, and is identical to the translation of **ȳ**. **ȳ** is decoded to produce **m̂**. The coloured bits in **m̂** correspond to the codons, and their translated amino acids, which are encoded in **ȳ**. Then **m̂** is translated back into alphanumeric form to give the original message. The Step by Step Embedding Section shows the individual encoding steps used to create **ȳ**.

Statistics of Sequences

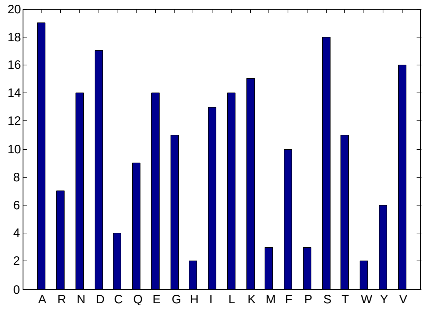

Figure A: Amino Acid count for $\bar{\mathbf{x}}$.

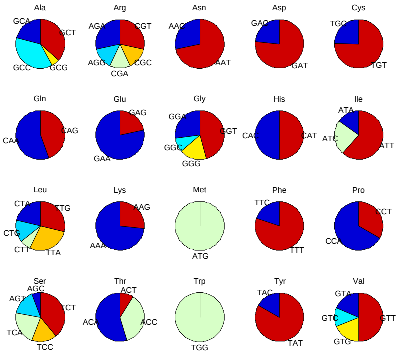

Figure B: Codon bias statistics of $\bar{\mathbf{x}}$.

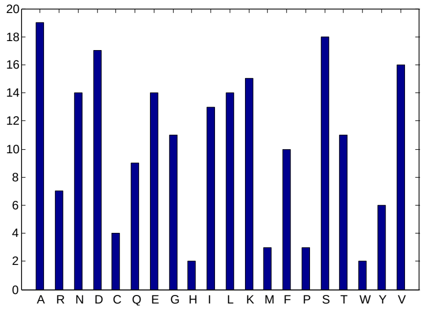

Figure C: Amino Acid count for $\bar{\mathbf{y}}$

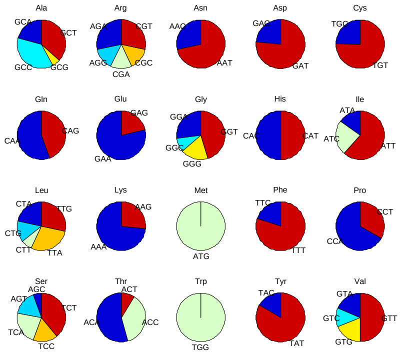

Figure D: Codon bias statistics of $\bar{\mathbf{y}}$.

Figure 2: Shown in the diagrams above is the statistics for the original host sequence $\bar{\mathbf{x}}$ and the encoded sequence $\bar{\mathbf{y}}$ from Figure 1. The amino acid translation is identical (Figures [A,C]), as it is with all other pcDNA data embedding algorithm. However BioCode pcDNA is the first algorithm to preserve the codon bias, as shown by Figures [B,D].

Step by Step Embedding

The following is the step by step procedure used to encode the information given in Figure 1. Only the current amino acid encoding table being used for each step is shown. In each of the frames below i is the current position in **a** (the amino acid sequence) being encoded. Amino acids, a_i, are represented by single letters given by Matlab's amino acid naming scheme and the full name of the amino acid is given in brackets beside this.

In the example below m is the current binary message to be embedded using the specified amino acid. When the amino acid a_i is given as an upper case calligraphic letter it denotes the set of codons which translate that amino acid, if a_i = R, then its corresponding set of codon which translate R is given by $\mathcal{R}$. This set is limited to the codons whose budget for usage has not been exhausted. The codon chosen for embedding is given by $\hat{\mathbf{y}}$. Once $\hat{\mathbf{y}}$ has been used for embedding the budget for that codon is reduced by one to give $c_{(\hat{\mathbf{y}})}$. When $c_{(\hat{\mathbf{y}})} = 0$ codon $\hat{\mathbf{y}}$ can no longer be used to encode data and $\hat{\mathbf{y}}$ is removed from the embedding table. Each codon translation set is mapped to a binary set to encode data. The first 80 encoding steps are shown below.

$i = 1$, a_1 = M (Methionine)

Cannot Embed

$m =$ —

$\hat{\mathbf{y}}$ = ATG

$i = 2$, a_2 = S (Serine)

Before Embedding

$m = 10$

$\mathcal{S}$	$\mathcal{M}_{\mathcal{S}}$
AGC	00
AGT	01
TCA	10
TCC	110
TCT	111

After Embedding

$\hat{\mathbf{y}}$ = TCA

$c_{(\hat{\mathbf{y}})} = 3$

$\mathcal{S}$	$\mathcal{M}_{\mathcal{S}}$
AGC	00
AGT	01
TCA	10
TCC	110
TCT	111

$i = 3$, a_3 = S (Serine)

Before Embedding

$m = 00$

$\mathcal{S}$	$\mathcal{M}_{\mathcal{S}}$
AGC	00
AGT	01
TCA	10
TCC	110
TCT	111

After Embedding

$\hat{\mathbf{y}}$ = AGC

$c_{(\hat{\mathbf{y}})} = 0$

$\mathcal{S}$	$\mathcal{M}_{\mathcal{S}}$
AGT	00
TCA	01
TCC	10
TCT	11

$i = 4$, a_4 = R (Arginine)

Before Embedding

$m = 01$

$\mathcal{R}$	$\mathcal{M}_{\mathcal{R}}$
AGA	00
AGG	01
CGA	10
CGC	110
CGT	111

After Embedding

$\hat{\mathbf{y}}$ = AGG

$c_{(\hat{\mathbf{y}})} = 0$

$\mathcal{R}$	$\mathcal{M}_{\mathcal{R}}$
AGA	00
CGA	01
CGC	10
CGT	11

$i = 5$, a_5 = K (Lysine)

Before Embedding

$m = 0$

$\mathcal{K}$	$\mathcal{M}_{\mathcal{K}}$
AAA	0
AAG	1

After Embedding

$\hat{\mathbf{y}}$ = AAA

$c_{(\hat{\mathbf{y}})} = 10$

$\mathcal{K}$	$\mathcal{M}_{\mathcal{K}}$
AAA	0
AAG	1

$i = 6$, a_6 = K (Lysine)

Before Embedding

$m = 1$

$\mathcal{K}$	$\mathcal{M}_{\mathcal{K}}$
AAA	0
AAG	1

After Embedding

$\hat{\mathbf{y}}$ = AAG

$c_{(\hat{\mathbf{y}})} = 3$

$\mathcal{K}$	$\mathcal{M}_{\mathcal{K}}$
AAA	0
AAG	1

$i = 7$, a_7 = N (Asparagine)

Before Embedding

$m = 1$

$\mathcal{N}$	$\mathcal{M}_{\mathcal{N}}$
AAC	0
AAT	1

After Embedding

$\hat{\mathbf{y}}$ = AAT
$c_{(\hat{\mathbf{y}})} = 9$

$\mathcal{N}$	$\mathcal{M}_{\mathcal{N}}$
AAC	0
AAT	1

$i = 8$, a_8 = I (Isoleucine)

Before Embedding

$m = 0$

$\mathcal{I}$	$\mathcal{M}_{\mathcal{I}}$
ATA	0
ATC	10
ATT	11

After Embedding

$\hat{\mathbf{y}}$ = ATA
$c_{(\hat{\mathbf{y}})} = 1$

$\mathcal{I}$	$\mathcal{M}_{\mathcal{I}}$
ATA	0
ATC	10
ATT	11

$i = 9$, a_9 = L (Leucine)

Before Embedding

$m = 10$

$\mathcal{L}$	$\mathcal{M}_{\mathcal{L}}$
CTA	00
CTG	01
CTT	10
TTA	110
TTG	111

After Embedding

$\hat{\mathbf{y}}$ = CTT
$c_{(\hat{\mathbf{y}})} = 0$

$\mathcal{L}$	$\mathcal{M}_{\mathcal{L}}$
CTA	00
CTG	01
TTA	10
TTG	11

$i = 10$, a_{10} = K (Lysine)

Before Embedding

$m = 0$

$\mathcal{K}$	$\mathcal{M}_{\mathcal{K}}$
AAA	0
AAG	1

After Embedding

$\hat{\mathbf{y}}$ = AAA
$c_{(\hat{\mathbf{y}})} = 9$

$\mathcal{K}$	$\mathcal{M}_{\mathcal{K}}$
AAA	0
AAG	1

$i = 11$, a_{11} = V (Valine)

Before Embedding

$m = 11$

$\mathcal{V}$	$\mathcal{M}_{\mathcal{V}}$
GTA	00
GTC	01
GTG	10
GTT	11

After Embedding

$\hat{\mathbf{y}}$ = GTT
$c_{(\hat{\mathbf{y}})} = 7$

$\mathcal{V}$	$\mathcal{M}_{\mathcal{V}}$
GTA	00
GTC	01
GTG	10
GTT	11

$i = 12$, a_{12} = I (Isoleucine)

Before Embedding

$m = 10$

$\mathcal{I}$	$\mathcal{M}_{\mathcal{I}}$
ATA	0
ATC	10
ATT	11

After Embedding

$\hat{\mathbf{y}}$ = ATC
$c_{(\hat{\mathbf{y}})} = 2$

$\mathcal{I}$	$\mathcal{M}_{\mathcal{I}}$
ATA	0
ATC	10
ATT	11

$i = 13$, a_{13} = I (Isoleucine)

Before Embedding

$m = 11$

$\mathcal{I}$	$\mathcal{M}_{\mathcal{I}}$
ATA	0
ATC	10
ATT	11

After Embedding

$\hat{\mathbf{y}}$ = ATT
$c_{(\hat{\mathbf{y}})} = 7$

$\mathcal{I}$	$\mathcal{M}_{\mathcal{I}}$
ATA	0
ATC	10
ATT	11

$i = 14$, a_{14} = L (Leucine)

Before Embedding

$m = 11$

$\mathcal{L}$	$\mathcal{M}_{\mathcal{L}}$
CTA	00
CTG	01
TTA	10
TTG	11

After Embedding

$\hat{\mathbf{y}}$ = TTG
$c_{(\hat{\mathbf{y}})} = 3$

$\mathcal{L}$	$\mathcal{M}_{\mathcal{L}}$
CTA	00
CTG	01
TTA	10
TTG	11

$i = 15$, a_{15} = G (Glycine)

Before Embedding

$m = 10$

$\mathcal{G}$	$\mathcal{M}_{\mathcal{G}}$
GGA	00
GGC	01
GGG	10
GGT	11

After Embedding

$\hat{\mathbf{y}}$ = GGG

$c_{(\hat{\mathbf{y}})} = 1$

$\mathcal{G}$	$\mathcal{M}_{\mathcal{G}}$
GGA	00
GGC	01
GGG	10
GGT	11

$i = 16$, a_{16} = D (Aspartic acid)

Before Embedding

$m = 0$

$\mathcal{D}$	$\mathcal{M}_{\mathcal{D}}$
GAC	0
GAT	1

After Embedding

$\hat{\mathbf{y}}$ = GAC

$c_{(\hat{\mathbf{y}})} = 3$

$\mathcal{D}$	$\mathcal{M}_{\mathcal{D}}$
GAC	0
GAT	1

$i = 17$, a_{17} = S (Serine)

Before Embedding

$m = 00$

$\mathcal{S}$	$\mathcal{M}_{\mathcal{S}}$
AGT	00
TCA	01
TCC	10
TCT	11

After Embedding

$\hat{\mathbf{y}}$ = AGT

$c_{(\hat{\mathbf{y}})} = 2$

$\mathcal{S}$	$\mathcal{M}_{\mathcal{S}}$
AGT	00
TCA	01
TCC	10
TCT	11

$i = 18$, a_{18} = G (Glycine)

Before Embedding

$m = 11$

$\mathcal{G}$	$\mathcal{M}_{\mathcal{G}}$
GGA	00
GGC	01
GGG	10
GGT	11

After Embedding

$\hat{\mathbf{y}}$ = GGT

$c_{(\hat{\mathbf{y}})} = 4$

$\mathcal{G}$	$\mathcal{M}_{\mathcal{G}}$
GGA	00
GGC	01
GGG	10
GGT	11

$i = 19$, a_{19} = V (Valine)

Before Embedding

$m = 11$

$\mathcal{V}$	$\mathcal{M}_{\mathcal{V}}$
GTA	00
GTC	01
GTG	10
GTT	11

After Embedding

$\hat{\mathbf{y}}$ = GTT

$c_{(\hat{\mathbf{y}})} = 6$

$\mathcal{V}$	$\mathcal{M}_{\mathcal{V}}$
GTA	00
GTC	01
GTG	10
GTT	11

$i = 20$, a_{20} = G (Glycine)

Before Embedding

$m = 01$

$\mathcal{G}$	$\mathcal{M}_{\mathcal{G}}$
GGA	00
GGC	01
GGG	10
GGT	11

After Embedding

$\hat{\mathbf{y}}$ = GGC

$c_{(\hat{\mathbf{y}})} = 0$

$\mathcal{G}$	$\mathcal{M}_{\mathcal{G}}$
GGA	0
GGG	10
GGT	11

$i = 21$, a_{21} = K (Lysine)

Before Embedding

$m = 1$

$\mathcal{K}$	$\mathcal{M}_{\mathcal{K}}$
AAA	0
AAG	1

After Embedding

$\hat{\mathbf{y}}$ = AAG

$c_{(\hat{\mathbf{y}})} = 2$

$\mathcal{K}$	$\mathcal{M}_{\mathcal{K}}$
AAA	0
AAG	1

$i = 22$, a_{22} = T (Threonine)

Before Embedding

$m = 11$

$\mathcal{T}$	$\mathcal{M}_{\mathcal{T}}$
ACA	0
ACC	10
ACT	11

After Embedding

$\hat{\mathbf{y}}$ = ACT

$c_{(\hat{\mathbf{y}})} = 0$

$\mathcal{T}$	$\mathcal{M}_{\mathcal{T}}$
ACA	0
ACC	1

$i = 23$, a_{23} = S (Serine)

Before Embedding

$m = 11$

$\mathcal{S}$	$\mathcal{M}_{\mathcal{S}}$
AGT	00
TCA	01
TCC	10
TCT	11

After Embedding

$\hat{\mathbf{y}}$ = TCT
$c_{(\hat{\mathbf{y}})} = 6$

$\mathcal{S}$	$\mathcal{M}_{\mathcal{S}}$
AGT	00
TCA	01
TCC	10
TCT	11

$i = 24$, a_{24} = L (Leucine)

Before Embedding

$m = 00$

$\mathcal{L}$	$\mathcal{M}_{\mathcal{L}}$
CTA	00
CTG	01
TTA	10
TTG	11

After Embedding

$\hat{\mathbf{y}}$ = CTA
$c_{(\hat{\mathbf{y}})} = 2$

$\mathcal{L}$	$\mathcal{M}_{\mathcal{L}}$
CTA	00
CTG	01
TTA	10
TTG	11

$i = 25$, a_{25} = M (Methionine)

Cannot Embed

$m = —$
$\hat{\mathbf{y}}$ = ATG

$i = 26$, a_{26} = H (Histidine)

Before Embedding

$m = 1$

$\mathcal{H}$	$\mathcal{M}_{\mathcal{H}}$
CAC	0
CAT	1

After Embedding

$\hat{\mathbf{y}}$ = CAT
$c_{(\hat{\mathbf{y}})} = 0$

Can no longer embed

$i = 27$, a_{27} = R (Arginine)

Before Embedding

$m = 00$

$\mathcal{R}$	$\mathcal{M}_{\mathcal{R}}$
AGA	00
CGA	01
CGC	10
CGT	11

After Embedding

$\hat{\mathbf{y}}$ = AGA
$c_{(\hat{\mathbf{y}})} = 1$

$\mathcal{R}$	$\mathcal{M}_{\mathcal{R}}$
AGA	00
CGA	01
CGC	10
CGT	11

$i = 28$, a_{28} = Y (Tyrosine)

Before Embedding

$m = 1$

$\mathcal{Y}$	$\mathcal{M}_{\mathcal{Y}}$
TAC	0
TAT	1

After Embedding

$\hat{\mathbf{y}}$ = TAT
$c_{(\hat{\mathbf{y}})} = 4$

$\mathcal{Y}$	$\mathcal{M}_{\mathcal{Y}}$
TAC	0
TAT	1

$i = 29$, a_{29} = V (Valine)

Before Embedding

$m = 10$

$\mathcal{V}$	$\mathcal{M}_{\mathcal{V}}$
GTA	00
GTC	01
GTG	10
GTT	11

After Embedding

$\hat{\mathbf{y}}$ = GTG
$c_{(\hat{\mathbf{y}})} = 2$

$\mathcal{V}$	$\mathcal{M}_{\mathcal{V}}$
GTA	00
GTC	01
GTG	10
GTT	11

$i = 30$, a_{30} = N (Asparagine)

Before Embedding

$m = 0$

$\mathcal{N}$	$\mathcal{M}_{\mathcal{N}}$
AAC	0
AAT	1

After Embedding

$\hat{\mathbf{y}}$ = AAC
$c_{(\hat{\mathbf{y}})} = 3$

$\mathcal{N}$	$\mathcal{M}_{\mathcal{N}}$
AAC	0
AAT	1

$i = 31$, $a_{31} = \mathrm{D}$ (Aspartic acid)

Before Embedding

$m = 1$

$\mathcal{D}$	$\mathcal{M}_{\mathcal{D}}$
GAC	0
GAT	1

After Embedding

$\hat{\mathbf{y}} = \mathrm{GAT}$
$c_{(\hat{\mathbf{y}})} = 12$

$\mathcal{D}$	$\mathcal{M}_{\mathcal{D}}$
GAC	0
GAT	1

$i = 32$, $a_{32} = \mathrm{K}$ (Lysine)

Before Embedding

$m = 0$

$\mathcal{K}$	$\mathcal{M}_{\mathcal{K}}$
AAA	0
AAG	1

After Embedding

$\hat{\mathbf{y}} = \mathrm{AAA}$
$c_{(\hat{\mathbf{y}})} = 8$

$\mathcal{K}$	$\mathcal{M}_{\mathcal{K}}$
AAA	0
AAG	1

$i = 33$, $a_{33} = \mathrm{Y}$ (Tyrosine)

Before Embedding

$m = 1$

$\mathcal{Y}$	$\mathcal{M}_{\mathcal{Y}}$
TAC	0
TAT	1

After Embedding

$\hat{\mathbf{y}} = \mathrm{TAT}$
$c_{(\hat{\mathbf{y}})} = 3$

$\mathcal{Y}$	$\mathcal{M}_{\mathcal{Y}}$
TAC	0
TAT	1

$i = 34$, $a_{34} = \mathrm{S}$ (Serine)

Before Embedding

$m = 01$

$\mathcal{S}$	$\mathcal{M}_{\mathcal{S}}$
AGT	00
TCA	01
TCC	10
TCT	11

After Embedding

$\hat{\mathbf{y}} = \mathrm{TCA}$
$c_{(\hat{\mathbf{y}})} = 2$

$\mathcal{S}$	$\mathcal{M}_{\mathcal{S}}$
AGT	00
TCA	01
TCC	10
TCT	11

$i = 35$, $a_{35} = \mathrm{Q}$ (Glutamine)

Before Embedding

$m = 0$

$\mathcal{Q}$	$\mathcal{M}_{\mathcal{Q}}$
CAA	0
CAG	1

After Embedding

$\hat{\mathbf{y}} = \mathrm{CAA}$
$c_{(\hat{\mathbf{y}})} = 4$

$\mathcal{Q}$	$\mathcal{M}_{\mathcal{Q}}$
CAA	0
CAG	1

$i = 36$, $a_{36} = \mathrm{Q}$ (Glutamine)

Before Embedding

$m = 0$

$\mathcal{Q}$	$\mathcal{M}_{\mathcal{Q}}$
CAA	0
CAG	1

After Embedding

$\hat{\mathbf{y}} = \mathrm{CAA}$
$c_{(\hat{\mathbf{y}})} = 3$

$\mathcal{Q}$	$\mathcal{M}_{\mathcal{Q}}$
CAA	0
CAG	1

$i = 37$, $a_{37} = \mathrm{Y}$ (Tyrosine)

Before Embedding

$m = 0$

$\mathcal{Y}$	$\mathcal{M}_{\mathcal{Y}}$
TAC	0
TAT	1

After Embedding

$\hat{\mathbf{y}} = \mathrm{TAC}$
$c_{(\hat{\mathbf{y}})} = 0$

Can no longer embed

$i = 38$, $a_{38} = \mathrm{K}$ (Lysine)

Before Embedding

$m = 0$

$\mathcal{K}$	$\mathcal{M}_{\mathcal{K}}$
AAA	0
AAG	1

After Embedding

$\hat{\mathbf{y}} = \mathrm{AAA}$
$c_{(\hat{\mathbf{y}})} = 7$

$\mathcal{K}$	$\mathcal{M}_{\mathcal{K}}$
AAA	0
AAG	1

$i = 39$, a_{39} = A (Alanine)

Before Embedding

$m = 01$

$\mathcal{A}$	$\mathcal{M}_\mathcal{A}$
GCA	00
GCC	01
GCG	10
GCT	11

After Embedding

$\hat{\mathbf{y}}$ = GCC
$c_{(\hat{\mathbf{y}})} = 6$

$\mathcal{A}$	$\mathcal{M}_\mathcal{A}$
GCA	00
GCC	01
GCG	10
GCT	11

$i = 40$, a_{40} = T (Threonine)

Before Embedding

$m = 1$

$\mathcal{T}$	$\mathcal{M}_\mathcal{T}$
ACA	0
ACC	1

After Embedding

$\hat{\mathbf{y}}$ = ACC
$c_{(\hat{\mathbf{y}})} = 3$

$\mathcal{T}$	$\mathcal{M}_\mathcal{T}$
ACA	0
ACC	1

$i = 41$, a_{41} = I (Isoleucine)

Before Embedding

$m = 10$

$\mathcal{I}$	$\mathcal{M}_\mathcal{I}$
ATA	0
ATC	10
ATT	11

After Embedding

$\hat{\mathbf{y}}$ = ATC
$c_{(\hat{\mathbf{y}})} = 1$

$\mathcal{I}$	$\mathcal{M}_\mathcal{I}$
ATA	0
ATC	10
ATT	11

$i = 42$, a_{42} = G (Glycine)

Before Embedding

$m = 0$

$\mathcal{G}$	$\mathcal{M}_\mathcal{G}$
GGA	0
GGG	10
GGT	11

After Embedding

$\hat{\mathbf{y}}$ = GGA
$c_{(\hat{\mathbf{y}})} = 2$

$\mathcal{G}$	$\mathcal{M}_\mathcal{G}$
GGA	0
GGG	10
GGT	11

$i = 43$, a_{43} = A (Alanine)

Before Embedding

$m = 00$

$\mathcal{A}$	$\mathcal{M}_\mathcal{A}$
GCA	00
GCC	01
GCG	10
GCT	11

After Embedding

$\hat{\mathbf{y}}$ = GCA
$c_{(\hat{\mathbf{y}})} = 3$

$\mathcal{A}$	$\mathcal{M}_\mathcal{A}$
GCA	00
GCC	01
GCG	10
GCT	11

$i = 44$, a_{44} = D (Aspartic acid)

Before Embedding

$m = 1$

$\mathcal{D}$	$\mathcal{M}_\mathcal{D}$
GAC	0
GAT	1

After Embedding

$\hat{\mathbf{y}}$ = GAT
$c_{(\hat{\mathbf{y}})} = 11$

$\mathcal{D}$	$\mathcal{M}_\mathcal{D}$
GAC	0
GAT	1

$i = 45$, a_{45} = F (Phenylalanine)

Before Embedding

$m = 1$

$\mathcal{F}$	$\mathcal{M}_\mathcal{F}$
TTC	0
TTT	1

After Embedding

$\hat{\mathbf{y}}$ = TTT
$c_{(\hat{\mathbf{y}})} = 7$

$\mathcal{F}$	$\mathcal{M}_\mathcal{F}$
TTC	0
TTT	1

$i = 46$, a_{46} = L (Leucine)

Before Embedding

$m = 10$

$\mathcal{L}$	$\mathcal{M}_\mathcal{L}$
CTA	00
CTG	01
TTA	10
TTG	11

After Embedding

$\hat{\mathbf{y}}$ = TTA
$c_{(\hat{\mathbf{y}})} = 3$

$\mathcal{L}$	$\mathcal{M}_\mathcal{L}$
CTA	00
CTG	01
TTA	10
TTG	11

$i = 47$, $a_{47} = $ T (Threonine)

Before Embedding

$m = 0$

$\mathcal{T}$	$\mathcal{M}_{\mathcal{T}}$
ACA	0
ACC	1

After Embedding

$\hat{\mathbf{y}} = $ ACA

$c_{(\hat{\mathbf{y}})} = 5$

$\mathcal{T}$	$\mathcal{M}_{\mathcal{T}}$
ACA	0
ACC	1

$i = 48$, $a_{48} = $ K (Lysine)

Before Embedding

$m = 1$

$\mathcal{K}$	$\mathcal{M}_{\mathcal{K}}$
AAA	0
AAG	1

After Embedding

$\hat{\mathbf{y}} = $ AAG

$c_{(\hat{\mathbf{y}})} = 1$

$\mathcal{K}$	$\mathcal{M}_{\mathcal{K}}$
AAA	0
AAG	1

$i = 49$, $a_{49} = $ E (Glutamic acid)

Before Embedding

$m = 0$

$\mathcal{E}$	$\mathcal{M}_{\mathcal{E}}$
GAA	0
GAG	1

After Embedding

$\hat{\mathbf{y}} = $ GAA

$c_{(\hat{\mathbf{y}})} = 10$

$\mathcal{E}$	$\mathcal{M}_{\mathcal{E}}$
GAA	0
GAG	1

$i = 50$, $a_{50} = $ V (Valine)

Before Embedding

$m = 11$

$\mathcal{V}$	$\mathcal{M}_{\mathcal{V}}$
GTA	00
GTC	01
GTG	10
GTT	11

After Embedding

$\hat{\mathbf{y}} = $ GTT

$c_{(\hat{\mathbf{y}})} = 5$

$\mathcal{V}$	$\mathcal{M}_{\mathcal{V}}$
GTA	00
GTC	01
GTG	10
GTT	11

$i = 51$, $a_{51} = $ T (Threonine)

Before Embedding

$m = 0$

$\mathcal{T}$	$\mathcal{M}_{\mathcal{T}}$
ACA	0
ACC	1

After Embedding

$\hat{\mathbf{y}} = $ ACA

$c_{(\hat{\mathbf{y}})} = 4$

$\mathcal{T}$	$\mathcal{M}_{\mathcal{T}}$
ACA	0
ACC	1

$i = 52$, $a_{52} = $ V (Valine)

Before Embedding

$m = 01$

$\mathcal{V}$	$\mathcal{M}_{\mathcal{V}}$
GTA	00
GTC	01
GTG	10
GTT	11

After Embedding

$\hat{\mathbf{y}} = $ GTC

$c_{(\hat{\mathbf{y}})} = 1$

$\mathcal{V}$	$\mathcal{M}_{\mathcal{V}}$
GTA	00
GTC	01
GTG	10
GTT	11

$i = 53$, $a_{53} = $ D (Aspartic acid)

Before Embedding

$m = 0$

$\mathcal{D}$	$\mathcal{M}_{\mathcal{D}}$
GAC	0
GAT	1

After Embedding

$\hat{\mathbf{y}} = $ GAC

$c_{(\hat{\mathbf{y}})} = 2$

$\mathcal{D}$	$\mathcal{M}_{\mathcal{D}}$
GAC	0
GAT	1

$i = 54$, $a_{54} = $ G (Glycine)

Before Embedding

$m = 11$

$\mathcal{G}$	$\mathcal{M}_{\mathcal{G}}$
GGA	0
GGG	10
GGT	11

After Embedding

$\hat{\mathbf{y}} = $ GGT

$c_{(\hat{\mathbf{y}})} = 3$

$\mathcal{G}$	$\mathcal{M}_{\mathcal{G}}$
GGA	0
GGG	10
GGT	11

$i = 55$, a_{55} = D (Aspartic acid)

Before Embedding

$m = 1$

$\mathcal{D}$	$\mathcal{M}_{\mathcal{D}}$
GAC	0
GAT	1

After Embedding

$\hat{\mathbf{y}}$ = GAT
$c_{(\hat{\mathbf{y}})}$ = 10

$\mathcal{D}$	$\mathcal{M}_{\mathcal{D}}$
GAC	0
GAT	1

$i = 56$, a_{56} = K (Lysine)

Before Embedding

$m = 1$

$\mathcal{K}$	$\mathcal{M}_{\mathcal{K}}$
AAA	0
AAG	1

After Embedding

$\hat{\mathbf{y}}$ = AAG
$c_{(\hat{\mathbf{y}})}$ = 0

Can no longer embed

$i = 57$, a_{57} = V (Valine)

Before Embedding

$m = 00$

$\mathcal{V}$	$\mathcal{M}_{\mathcal{V}}$
GTA	00
GTC	01
GTG	10
GTT	11

After Embedding

$\hat{\mathbf{y}}$ = GTA
$c_{(\hat{\mathbf{y}})}$ = 2

$\mathcal{V}$	$\mathcal{M}_{\mathcal{V}}$
GTA	00
GTC	01
GTG	10
GTT	11

$i = 58$, a_{58} = A (Alanine)

Before Embedding

$m = 11$

$\mathcal{A}$	$\mathcal{M}_{\mathcal{A}}$
GCA	00
GCC	01
GCG	10
GCT	11

After Embedding

$\hat{\mathbf{y}}$ = GCT
$c_{(\hat{\mathbf{y}})}$ = 6

$\mathcal{A}$	$\mathcal{M}_{\mathcal{A}}$
GCA	00
GCC	01
GCG	10
GCT	11

$i = 59$, a_{59} = T (Threonine)

Before Embedding

$m = 1$

$\mathcal{T}$	$\mathcal{M}_{\mathcal{T}}$
ACA	0
ACC	1

After Embedding

$\hat{\mathbf{y}}$ = ACC
$c_{(\hat{\mathbf{y}})}$ = 2

$\mathcal{T}$	$\mathcal{M}_{\mathcal{T}}$
ACA	0
ACC	1

$i = 60$, a_{60} = M (Methionine)

Cannot Embed

m = —
$\hat{\mathbf{y}}$ = ATG

$i = 61$, a_{61} = Q (Glutamine)

Before Embedding

$m = 1$

$\mathcal{Q}$	$\mathcal{M}_{\mathcal{Q}}$
CAA	0
CAG	1

After Embedding

$\hat{\mathbf{y}}$ = CAG
$c_{(\hat{\mathbf{y}})}$ = 3

$\mathcal{Q}$	$\mathcal{M}_{\mathcal{Q}}$
CAA	0
CAG	1

$i = 62$, a_{62} = V (Valine)

Before Embedding

$m = 00$

$\mathcal{V}$	$\mathcal{M}_{\mathcal{V}}$
GTA	00
GTC	01
GTG	10
GTT	11

After Embedding

$\hat{\mathbf{y}}$ = GTA
$c_{(\hat{\mathbf{y}})}$ = 1

$\mathcal{V}$	$\mathcal{M}_{\mathcal{V}}$
GTA	00
GTC	01
GTG	10
GTT	11

$i = 63$, a_{63} = W (Tryptophan)

Cannot Embed

$m = —$
$\hat{\mathbf{y}}$ = TGG

$i = 65$, a_{65} = T (Threonine)

Before Embedding

$m = 0$

$\mathcal{T}$	$\mathcal{M}_{\mathcal{T}}$
ACA	0
ACC	1

After Embedding

$\hat{\mathbf{y}}$ = ACA
$c_{(\hat{\mathbf{y}})}$= 3

$\mathcal{T}$	$\mathcal{M}_{\mathcal{T}}$
ACA	0
ACC	1

$i = 67$, a_{67} = G (Glycine)

Before Embedding

$m = 11$

$\mathcal{G}$	$\mathcal{M}_{\mathcal{G}}$
GGA	0
GGG	10
GGT	11

After Embedding

$\hat{\mathbf{y}}$ = GGT
$c_{(\hat{\mathbf{y}})}$= 2

$\mathcal{G}$	$\mathcal{M}_{\mathcal{G}}$
GGA	0
GGG	10
GGT	11

$i = 69$, a_{69} = E (Glutamic acid)

Before Embedding

$m = 0$

$\mathcal{E}$	$\mathcal{M}_{\mathcal{E}}$
GAA	0
GAG	1

After Embedding

$\hat{\mathbf{y}}$ = GAA
$c_{(\hat{\mathbf{y}})}$= 9

$\mathcal{E}$	$\mathcal{M}_{\mathcal{E}}$
GAA	0
GAG	1

$i = 64$, a_{64} = D (Aspartic acid)

Before Embedding

$m = 1$

$\mathcal{D}$	$\mathcal{M}_{\mathcal{D}}$
GAC	0
GAT	1

After Embedding

$\hat{\mathbf{y}}$ = GAT
$c_{(\hat{\mathbf{y}})}$= 9

$\mathcal{D}$	$\mathcal{M}_{\mathcal{D}}$
GAC	0
GAT	1

$i = 66$, a_{66} = A (Alanine)

Before Embedding

$m = 11$

$\mathcal{A}$	$\mathcal{M}_{\mathcal{A}}$
GCA	00
GCC	01
GCG	10
GCT	11

After Embedding

$\hat{\mathbf{y}}$ = GCT
$c_{(\hat{\mathbf{y}})}$= 5

$\mathcal{A}$	$\mathcal{M}_{\mathcal{A}}$
GCA	00
GCC	01
GCG	10
GCT	11

$i = 68$, a_{68} = Q (Glutamine)

Before Embedding

$m = 0$

$\mathcal{Q}$	$\mathcal{M}_{\mathcal{Q}}$
CAA	0
CAG	1

After Embedding

$\hat{\mathbf{y}}$ = CAA
$c_{(\hat{\mathbf{y}})}$= 2

$\mathcal{Q}$	$\mathcal{M}_{\mathcal{Q}}$
CAA	0
CAG	1

$i = 70$, a_{70} = R (Arginine)

Before Embedding

$m = 10$

$\mathcal{R}$	$\mathcal{M}_{\mathcal{R}}$
AGA	00
CGA	01
CGC	10
CGT	11

After Embedding

$\hat{\mathbf{y}}$ = CGC
$c_{(\hat{\mathbf{y}})}$= 0

$\mathcal{R}$	$\mathcal{M}_{\mathcal{R}}$
AGA	0
CGA	10
CGT	11

$i = 71$, $a_{71} =$ F (Phenylalanine)

Before Embedding

$m = 1$

$\mathcal{F}$	$\mathcal{M}_{\mathcal{F}}$
TTC	0
TTT	1

After Embedding

$\hat{\mathbf{y}}$ = TTT
$c_{(\hat{\mathbf{y}})} = 6$

$\mathcal{F}$	$\mathcal{M}_{\mathcal{F}}$
TTC	0
TTT	1

$i = 73$, $a_{73} =$ S (Serine)

Before Embedding

$m = 10$

$\mathcal{S}$	$\mathcal{M}_{\mathcal{S}}$
AGT	00
TCA	01
TCC	10
TCT	11

After Embedding

$\hat{\mathbf{y}}$ = TCC
$c_{(\hat{\mathbf{y}})} = 2$

$\mathcal{S}$	$\mathcal{M}_{\mathcal{S}}$
AGT	00
TCA	01
TCC	10
TCT	11

$i = 75$, $a_{75} =$ G (Glycine)

Before Embedding

$m = 0$

$\mathcal{G}$	$\mathcal{M}_{\mathcal{G}}$
GGA	0
GGG	10
GGT	11

After Embedding

$\hat{\mathbf{y}}$ = GGA
$c_{(\hat{\mathbf{y}})} = 1$

$\mathcal{G}$	$\mathcal{M}_{\mathcal{G}}$
GGA	0
GGG	10
GGT	11

$i = 77$, $a_{77} =$ A (Alanine)

Before Embedding

$m = 00$

$\mathcal{A}$	$\mathcal{M}_{\mathcal{A}}$
GCA	00
GCC	01
GCG	10
GCT	11

After Embedding

$\hat{\mathbf{y}}$ = GCA
$c_{(\hat{\mathbf{y}})} = 2$

$\mathcal{A}$	$\mathcal{M}_{\mathcal{A}}$
GCA	00
GCC	01
GCG	10
GCT	11

$i = 72$, $a_{72} =$ Q (Glutamine)

Before Embedding

$m = 1$

$\mathcal{Q}$	$\mathcal{M}_{\mathcal{Q}}$
CAA	0
CAG	1

After Embedding

$\hat{\mathbf{y}}$ = CAG
$c_{(\hat{\mathbf{y}})} = 2$

$\mathcal{Q}$	$\mathcal{M}_{\mathcal{Q}}$
CAA	0
CAG	1

$i = 74$, $a_{74} =$ L (Leucine)

Before Embedding

$m = 11$

$\mathcal{L}$	$\mathcal{M}_{\mathcal{L}}$
CTA	00
CTG	01
TTA	10
TTG	11

After Embedding

$\hat{\mathbf{y}}$ = TTG
$c_{(\hat{\mathbf{y}})} = 2$

$\mathcal{L}$	$\mathcal{M}_{\mathcal{L}}$
CTA	00
CTG	01
TTA	10
TTG	11

$i = 76$, $a_{76} =$ V (Valine)

Before Embedding

$m = 11$

$\mathcal{V}$	$\mathcal{M}_{\mathcal{V}}$
GTA	00
GTC	01
GTG	10
GTT	11

After Embedding

$\hat{\mathbf{y}}$ = GTT
$c_{(\hat{\mathbf{y}})} = 4$

$\mathcal{V}$	$\mathcal{M}_{\mathcal{V}}$
GTA	00
GTC	01
GTG	10
GTT	11

$i = 78$, $a_{78} =$ F (Phenylalanine)

Before Embedding

$m = 1$

$\mathcal{F}$	$\mathcal{M}_{\mathcal{F}}$
TTC	0
TTT	1

After Embedding

$\hat{\mathbf{y}}$ = TTT
$c_{(\hat{\mathbf{y}})} = 5$

$\mathcal{F}$	$\mathcal{M}_{\mathcal{F}}$
TTC	0
TTT	1

$i = 79$, $a_{79} = $ Y (Tyrosine)

Cannot Embed

$m = $ —
$\hat{\mathbf{y}} = $ TAT

$i = 80$, $a_{80} = $ R (Arginine)

Before Embedding

$m = 0$

$\mathcal{R}$	$\mathcal{M}_{\mathcal{R}}$
AGA	0
CGA	10
CGT	11

After Embedding

$\hat{\mathbf{y}} = $ AGA
$c_{(\hat{\mathbf{y}})} = 0$

$\mathcal{R}$	$\mathcal{M}_{\mathcal{R}}$
CGA	0
CGT	1
